# Targeting KIT prevents brain arteriovenous malformations driven by ALK1-deficient angiogenic endothelial cells

**DOI:** 10.1172/JCI197587

**Published:** 2026-08-11

**Authors:** Elise Drapé, Lauranne Carrier, Gael Cagnone, Atik Rohmana Maftuhatul Fuad, Mathilde Bizou, Damian Sanchez, Typhaine Anquetil, Jack Wang, Halima Drissi Touzani Walali, Adnan Gopinadhan, Patrick Piet van Vliet, Joel P. Howard, Mysha Ibnat, Gregor Andelfinger, Ethan Winkler, Bruno Larrivée, Alexandre Dubrac

**Affiliations:** 1 Département De Pharmacologie Et De Physiologie, Faculté de médecine, Université de Montréal, Montréal, Centre De Recherche CHU St. Justine, Montréal, Quebec, Canada.; 2 Centre De Recherche CHU St. Justine, Montréal, Quebec, Canada.; 3 Département De Biochimie, Faculté de médecine, Université de Montréal, Centre De Recherche CHU St. Justine, Montréal, Quebec, Canada.; 4 Department of Anatomy and Cell Biology, School of Biomedical Sciences, McGill University, Montréal, Quebec, Canada.; 5 Département De Pathologie Et Biologie Cellulaire, Faculté de médecine, Université de Montréal, Centre De Recherche CHU St. Justine, Montréal, Quebec, Canada.; 6 Department of Neurological Surgery, University of California, San Francisco, San Francisco, California, USA.; 7 Département d’Ophtalmologie, Faculté de médecine, Université de Montréal, Centre de Recherche de l’Hôpital Maisonneuve-Rosemont, Montréal, Quebec, Canada.

**Keywords:** Cell biology, Vascular biology, Angiogenesis, Endothelial cells, Transcriptomics

## Abstract

Hereditary hemorrhagic telangiectasia type 2 (HHT2), caused by mutations in *ACVRL1* (also known as *ALK1*), is characterized by brain arteriovenous malformations (bAVMs), abnormal artery–vein connections for which treatment options remain limited. Despite evidence of endothelial cell (EC) heterogeneity, its role in bAVM pathogenesis remains poorly defined. Using EC-specific inducible *Alk1*-knockout mice (*Alk1iECKO*) and regionally resolved single-cell RNA sequencing, we showed that loss of ALK1 signaling induces bAVMs predominantly in the perineural vascular plexus. This process is driven by the emergence of a KIT^+^ angiogenic EC population with human AVM-like transcriptional features, including tip cell markers and activation of PI3K and KRAS signaling pathways. Cross-species analyses and validation in human samples demonstrated that KIT expression is conserved in ECs from both sporadic and HHT2 bAVMs. Drug-repurposing analysis identified KIT as a top actionable target, and we showed that *Kit* is directly repressed by BMP9/ALK1/SMAD4 signaling. Pharmacological inhibition of KIT reduced angiogenic reprogramming and vascular malformations in vivo without affecting normal vasculature. These findings identify a pathogenic angiogenic EC state and position KIT signaling as a therapeutically actionable pathway in bAVMs.

## Abstract 2

Loss of ALK1 signaling drives brain vessel malformations via KIT⁺ angiogenic endothelial cells. Targeting KIT with approved drugs prevents these lesions in a mouse model.

## Graphical abstract

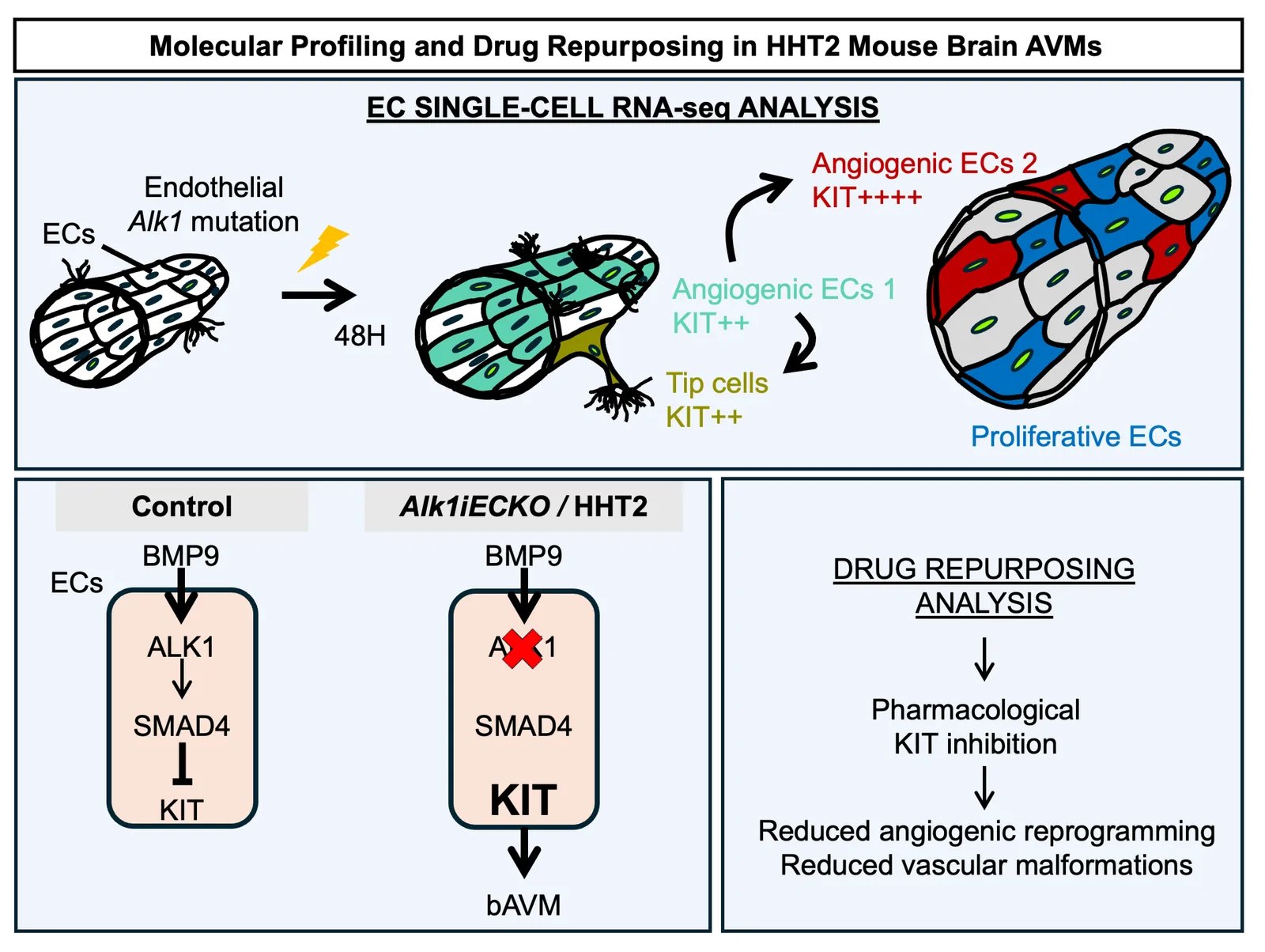

## Introduction

Hereditary hemorrhagic telangiectasia (HHT), or Rendu-Osler syndrome, is a rare genetic disorder affecting 1 in 5,000 to 8,000 individuals worldwide (1, 2). It is caused by mutations in components of the BMP9/ALK1 signaling pathway, which regulates vascular development and homeostasis. Mutations in the *ENG* and *ACVRL1* genes, encoding ENDOGLIN and ALK1, respectively, account for the majority of HHT cases (HHT1 and HHT2) (3–5). Rare mutations in *GDF2* or *SMAD4* result in HHT5 and juvenile polyposis-HHT (6, 7). Patients with HHT develop recurrent epistaxis, telangiectasia, and arteriovenous malformations (AVMs) in various organs, with brain AVMs (bAVMs) occurring in 10%–20% of cases (8, 9). AVMs are vascular anomalies characterized by the direct connection of arteries and veins without an intervening capillary bed. These lesions are associated with severe complications, including epilepsy and cerebral hemorrhage (10, 11). Current management of bAVMs relies on invasive procedures, including microsurgical resection, endovascular embolization, and stereotactic radiosurgery, which are associated with substantial risks. Despite ongoing efforts to develop pharmacological approaches targeting pathways such as VEGF, BMP9/ALK1 signaling, tyrosine kinases, and PI3K/mTOR, no medical therapy is currently approved for bAVMs, underscoring the need for new therapeutic strategies (12–17).

The pathogenesis of bAVM remains poorly understood. BMP9 and BMP10 bind ALK1 and its coreceptors, including BMPRII and ENDOGLIN, activating SMAD1/5/8 and SMAD4 transcription factors. Studies using different HHT mouse models have highlighted that deletions of ALK1, ENDOGLIN, SMAD1/5, or SMAD4 in endothelial cells (ECs) are sufficient to induce bAVMs (18–21). Moreover, recent studies have demonstrated that the deletion of *ALK1* and *SMAD4* specifically in venous ECs induces AVM formations, whereas similar deletions in arterial ECs do not, suggesting that AVMs originate primarily from venous ECs (22, 23). Loss of BMP9/ALK1/SMAD4 signaling modulates the response of ECs to shear stress, activating VEGF-induced PI3K/AKT signaling and the integrin/YAP/TAZ pathways (14, 20, 22–24). As a result, mutant ECs display hyperproliferation, loss of arteriovenous identity, and impaired migration against blood flow (20, 22, 23, 25, 26). However, most mechanistic insights have been derived from postnatal retinal models, while the specific cellular and molecular mechanisms in bAVMs remain poorly understood. Moreover, these findings suggest the BMP9/ALK1/SMAD4 signaling disruptions alter the transcriptomic landscape of ECs, driving cellular heterogeneity and contributing to the diverse phenotypes observed in AVMs.

In addition to these molecular mechanisms, EC heterogeneity has emerged as a defining feature of AVMs, including those in the brain. This heterogeneity encompasses differences in cellular identity, metabolic activity, and proliferative states, all of which contribute to the distinct structural and functional abnormalities of AVMs. Pathological endothelial plasticity, characterized by proliferative ECs with mixed arteriovenous (AV) characteristics, disrupts physiological arteriovenous zonation. Recent studies utilizing single-cell transcriptomics have provided critical insights into this heterogeneity. For instance, Winkler and colleagues (27) identified a distinct subset of ECs in bAVMs exhibiting heightened angiogenic and immunogenic potential and originating from the AVM nidus. Moreover, this pathological remodeling involves alterations in CNS-specific vascular properties coupled with the reactivation of developmental pathways (28). Similarly, a recent study in retinal *Alk1*-mutant mice has shown that some ECs within AVMs exhibit enhanced proliferation and mixed AV characteristics (29). Yang et al. (30) further described arterial-lymphatic-like ECs arising from arterial ECs after *Alk1* deletion, implicating aberrant plasticity in lung AVM development. However, the specific EC populations driving HHT AVM pathogenesis in the brain and retina remain undefined. Identifying and characterizing ECs driving AVM is essential for understanding AVM pathogenesis and developing effective therapeutic strategies.

Here, we investigate the contribution of EC heterogeneity to bAVM pathogenesis in HHT2 using an EC-specific *Alk1*-knockout mouse model combined with single-cell transcriptomics. We identify distinct angiogenic EC populations, including a potentially previously uncharacterized cluster that emerges specifically in the brain surface vasculature and exhibits transcriptomic similarity to human bAVM ECs. Among the defining features of this population, we identify KIT as a direct downstream target of BMP9/ALK1 signaling. Moreover, pharmacological KIT signaling inhibition limits angiogenic reprogramming and vascular malformations in vivo. Our findings highlight a distinct angiogenic EC population as a key driver of AVM formation in HHT2 and identify KIT as a candidate therapeutic target for bAVMs.

## Results

### Brain endothelial Alk1 deletion induces region-specific AVMs in the postnatal brain.

To investigate bAVM formation in a model of HHT2, we used EC-specific and inducible *Alk1*-knockout (*Alk1iECKO*) mice. Tamoxifen was injected at P6, and the vascular phenotypes were assessed at P8 to circumvent early lethality observed in this model (29), which we quantified and found to occur approximately 48–50 hours after tamoxifen injection (Figure 1A and Supplemental Figure 1A; supplemental material available online with this article; https://doi.org/10.1172/JCI197587DS1). This time point corresponds to an active phase of postnatal brain angiogenesis, AV maturation, and vascular remodeling (31, 32), allowing efficient induction of AVM formation upon *Alk1* deletion.

We analyzed the PNVP and INVP as distinct vascular compartments because of their different vessel compositions and hemodynamic environments (33) (Figure 1A). The PNVP, located at the brain surface, is primarily composed of veins, venules, and arteries and undergoes active remodeling during early postnatal development, including a progressive decrease in vascular density during the first 2 postnatal weeks. It exhibits higher flow patterns compared with the INVP, which is largely composed of capillary beds within the brain parenchyma. Given that AVMs arise from aberrant AV connections and are strongly influenced by flow, we hypothesized that the PNVP would be more susceptible to bAVM formation upon loss of ALK1 signaling.

To examine the vascular network, we performed immunostaining for the endothelial marker CD31 on whole-mount brain preparations to assess the PNVP and on coronal brain sections to examine the INVP (Figure 1A and Supplemental Figure 1B). To distinguish arteries within the vascular network, we costained with an antibody against α-SMA, a marker of vascular smooth muscle cells (34) (Figure 1A and Supplemental Figure 1B). We observed that loss of ALK1 signaling induced vascular malformations in the PNVP compared with control littermates at P8 (Figure 1B). Specifically, we detected a significant increase in the overall vascular area (Figure 1C). This change was associated with a significant increase in the diameter of arteries, veins, and venules (Figure 1D), along with a marked reduction in the proportion of CD31^+^ vessels smaller than 10 μm and a corresponding shift toward larger vessel diameters (>10 μm), although these changes did not reach statistical significance (Figure 1E).

In line with previous reports that identified abnormal vein filling with blue latex in the presence of AV shunts (24), we also observed direct AV connections in the mutant PNVP, indicative of early bAVM formation (Figure 1F). This phenotype was accompanied by a significant increase in both the number and diameter of PNVP AV connections (Figure 1, G and H). While we confirmed efficient *Alk1* deletion in ECs isolated from both the PNVP and INVP compartments and validated loss of ALK1 protein in INVP vessels (Supplemental Figure 1, C and D), we did not observe marked vascular alterations in the INVP. Specifically, vessel diameter and vascular area remained unchanged in the mutant INVP compared with controls (Figure 1, I–K). These results indicate that a 48-hour loss of endothelial ALK1 signaling predominantly induces vascular malformations and AVMs in the PNVP.

### Single-cell RNA sequencing reveals that Alk1 deficiency induces EC transcriptomic aberrancy in PNVP and INVP ECs.

To investigate the molecular mechanisms driving AVM formation in the PNVP, we performed single-cell RNA sequencing (scRNA-seq) of ECs isolated from both PNVP and INVP regions of the same brains in P8 *Alk1iECKO* and control mice (Supplemental Figure 2A). We sequenced 1,195 *Alk1l/l* and 2,193 *Alk1iECKO* PNVP ECs and 3,221 *Alk1l/l* and 1,904 *Alk1iECKO* INVP ECs, with an average of 4,097 genes per cell.

Uniform manifold approximation and projection (UMAP) visualization and unsupervised clustering revealed distinct EC subpopulations in both genotypes and brain regions (Figure 2A and Supplemental Figure 2B). In the INVP of control mice, we identified a typical continuum of arterial to venous ECs, with a predominance of capillary ECs, as well as proliferative and tip cell populations reflecting the active angiogenic phase of postnatal development. In contrast, the PNVP of control brains was mostly composed of venous, venular, and arterial ECs, consistent with a vascular bed undergoing hemodynamic remodeling at this stage (Figure 2, A and B). Moreover, in the PNVP dataset, we also detected capillary and tip ECs resembling those in the INVP, which could reflect partial contamination because of enzymatic overdigestion of the cortical INVP during PNVP EC isolation. Nonetheless, the major EC populations identified across samples were consistent with previously described vascular zones and endothelial subtype identities (35).

To confirm efficient deletion of *Alk1*, we performed pathway activity score analysis (VISION package) using a custom ALK1 signaling signature gene set (36) (Supplemental Table 1). This revealed a marked reduction in ALK1 target gene expression across all mutant EC clusters, in both PNVP and INVP (Figure 2, C and D). In the PNVP, *Alk1iECKO* ECs showed a striking loss of arterial, venular, and venous identity, accompanied by the emergence of new arterial-like, venular-like, and venous-like clusters (Figure 2, E and F, and Supplemental Figure 2, C and D). Similarly, though less pronounced, loss of AV identities was also observed in the INVP.

Notably, we observed that *Alk1* deletion led to increased EC proliferation predominantly in the PNVP, consistent with the vascular malformations seen in vivo (Figure 1, B–K, and Figure 2, A and B). This was supported by EdU (5-ethynyl-2′-deoxyuridine)/ERG1/2/3 (ETS-related gene) colabeling experiments, which showed a significant increase in proliferating ECs specifically in the mutant PNVP (Figure 2, G and H).

Given prior studies suggesting blood-brain barrier (BBB) defects in AVMs (37–39), we also assessed BBB-related gene expression. Using a custom BBB gene set (Supplemental Table 1), pathway activity analysis indicated that while the INVP normally displays a stronger BBB signature than the PNVP, loss of ALK1 signaling resulted in a more pronounced BBB gene downregulation in the PNVP (Supplemental Figure 2, E and F), highlighting region-specific vascular vulnerabilities in P8 *Alk1iECKO* brain.

Importantly, we performed an independent biological replicate of the scRNA-seq experiment, which yielded consistent endothelial clustering and marker gene expression patterns (Supplemental Figure 3A).

### Characterization of PNVP-enriched angiogenic EC clusters in Alk1iECKO brains.

BMP9/ALK1 signaling is a key regulator of angiogenic sprouting and tip cell specification (36). Consistent with previous findings in the retina, *Alk1* deletion resulted in an increased number of tip ECs in the brain (Figure 3, A–C). In addition, it induced the emergence of 2 angiogenic endothelial clusters in both the INVP and PNVP regions (Figure 2, A and B, and Figure 3A), annotated as angiogenic 1 and angiogenic 2 clusters based on the expression of tip EC markers (Figure 3D), consistent with the heightened angiogenic activity characteristic of AVMs (40).

While the angiogenic 1 ECs exhibited strong transcriptomic similarity to canonical tip cells, angiogenic 2 ECs displayed a divergent expression profile marked by genes such as *Slc16a3*, *Pgf*, and *Stc1* (Figure 3, E and F, and Supplemental Figure 3B). Differential pathway activity analysis revealed that angiogenic 1 ECs displayed upregulation of pathways involved in cardiac contraction and regulation of blood circulation, whereas angiogenic 2 ECs were enriched in pathways related to glycolysis and hypoxia (Supplemental Figure 4, A and B). Focusing on the hallmark pathways from Molecular Signatures Database (MSigDB), the angiogenic 2 cluster also exhibited increased enrichment for several bAVM-related gene signatures compared with other EC clusters, particularly relative to the angiogenic 1 cluster (Figure 3G). These included pathways previously reported in human bAVMs, such as inflammatory response, epithelial-to-mesenchymal transition, complement, angiogenesis, and PI3K/mTOR signaling (27). We also noted dysregulation of KRAS and NOTCH signaling, both implicated in AVM pathogenesis (14). Moreover, angiogenic 2 ECs expressed genes previously reported to be upregulated in *Alk1*-deficient ECs, such as *Itgb1* and *Itgav* (22), and genes recently identified as candidate drivers in HHT, including *Jak2*, *Angptl4*, and *Foxo1* (41) (Supplemental Figure 4C). These data support functionally divergent angiogenic states shaped by tissue context: angiogenic 1 ECs predominated in the INVP, replacing capillary ECs, whereas angiogenic 2 ECs were selectively enriched in the PNVP.

Given that AVMs predominantly form in the PNVP at P8, we hypothesized that the angiogenic 2 cluster represents ECs within AVMs. Supporting this, in situ hybridization (ISH) for *Stc1* confirmed increased expression in PNVP ECs of *Alk1iECKO* mice (Figure 3H and Supplemental Figure 4D), consistent with angiogenic 2 ECs comprising the AVM endothelium.

Because AVMs are believed to originate from the venous vascular bed (22, 23), we used vein cluster as a root for single-cell trajectory analysis, allowing pseudotime inference based on transcriptomic variations across cells, which can then be visualized on UMAP dimensions. In control brains, venous ECs transitioned toward proliferative or capillary ECs, which then gave rise to arterial ECs, consistent with known postnatal vascular maturation (Figure 3I). In contrast, in *Alk1iECKO* ECs, venous ECs progressed to angiogenic 1 ECs and subsequently gave rise to tip cells and angiogenic 2 ECs (Figure 3I).

These data support a venous origin for AVMs and suggest that angiogenic 1 ECs represent an early reprogrammed state that can evolve into angiogenic 2 ECs. Together, our results point to angiogenic 2 ECs as a transcriptomic signature of AVM-forming endothelium.

### Mouse Alk1iECKO angiogenic 2 and human bAVM endothelial clusters share AVM molecular markers, including KIT.

To explore the transcriptional overlap between the angiogenic 2 cluster in *Alk1iECKO* brains and endothelial clusters identified in human bAVMs by Winkler E. et al. (27), we next examined shared molecular markers. Cross-species comparison revealed common upregulated genes involved in angiogenesis, including *PGF*, *STC2*, *APLN*, and *KIT* (Figure 4, A–C, and Supplemental Figure 5, A and B). We also found these markers enriched in angiogenic capillary ECs from another human bAVM dataset published by Wälchli et al. (28) (Supplemental Figure 5C), suggesting conserved human AVM-associated endothelial reprogramming in mouse *Alk1iECKO* brains.

To characterize the vascular communication profile of these AVM-associated clusters, we performed ligand–receptor connectome analysis based on ligand–receptor expression levels using the Connectome R package (42), examining signaling interactions of the *Alk1iECKO* angiogenic 2 cluster and the human AVM nidus cluster with other EC populations (Figure 4D and Supplemental Figure 5D). Both clusters exhibited widespread interactions with other EC types, primarily through angiogenic, integrin, and ECM-related signals, such as *Itgb1*, *Pgf*, and *Kit* (Figure 4D and Supplemental Figure 5D). Surprisingly, we detected very few ligand–receptor interactions between angiogenic 2 and proliferative EC clusters, despite both being present in the mouse AVM dataset (Winkler et al. [ref. 27] did not annotate proliferative clusters in their human AVM dataset).

Taken together, the prominence of *Kit* among the shared angiogenic genes, and its predicted ligand–receptor interactions, collectively highlight KIT as a compelling and targetable vulnerability of the angiogenic 2 state. *Kit* encodes an RTK activated by its ligand, stem cell factor (encoded by *Kitl*), which regulates cell migration and proliferation in immune and cancer contexts (43, 44). KIT is also an attractive candidate for therapeutic targeting, as KIT inhibitors are widely used in oncology, suggesting potential for drug repurposing if KIT signaling contributes to bAVM pathogenesis (45).

Pathway activity analysis revealed robust enrichment of KIT signaling in the angiogenic 2 cluster, with a lower increase in angiogenic 1, consistent with a potential progression from angiogenic 1 to angiogenic 2 ECs (Figure 4E). In our dataset, *Kitl* was broadly expressed across EC clusters, with highest levels in arterial ECs and lowest in venous ECs, while *Kit* showed an inverse gradient, low in venous ECs and absent in arterial ECs (Figure 4F). In control ECs, *Kit* expression was largely restricted to tip cells, whereas in mutant ECs, it was broadly upregulated in angiogenic EC populations, with predominant enrichment in the angiogenic 2 cluster. Notably, both *Kit* and *Kitl* were coexpressed in angiogenic 2 ECs, supporting the possibility of cell-autonomous KIT activation, consistent with their predicted Connectome interactions (Figure 4, D–F).

We then validated the upregulation of *Kit* mRNA in *Alk1iECKO* ECs using ISH, which confirmed strong expression in INVP tip cells and a mosaic increase in a subset of PNVP ECs, consistent with its specific enrichment in the angiogenic 2 cluster (Figure 4, G and H). This upregulation appeared brain specific, as *Kit* expression remained unchanged in lung ECs isolated from *Alk1iECKO* mice (Supplemental Figure 5E). Importantly, KIT protein expression was confirmed by immunostaining in human HHT2 bAVM endothelium (Figure 4I) and was also enriched in ECs from sporadic human bAVMs (Supplemental Figure 6).

### ALK1 signaling suppresses KIT and angiogenic 2 gene expression in vitro.

We next investigated whether BMP9/ALK1 signaling directly regulates KIT expression in ECs. HUVECs were transfected with either control or *ALK1* (*Acvrl1*) siRNA, treated for 24 hours with 5 ng/mL BMP9, and analyzed for KIT expression at both the protein and mRNA levels using Western blotting and RT-qPCR, respectively. Efficient *ALK1* knockdown in HUVECs was first confirmed by qPCR (Supplemental Figure 7A). In control-transfected cells, KIT was expressed at high levels under basal conditions, but BMP9 treatment robustly induced phosphorylated (p-) SMAD1/5/9 and led to a strong reduction of KIT expression at both the protein and mRNA levels (Figure 5, A–C). Silencing *ALK1* prevented BMP9-induced SMAD1/5/9 phosphorylation and rescued KIT expression, confirming the requirement of ALK1 for BMP9-mediated KIT repression (Figure 5, A–C).

We found similar results in human brain endothelial cells (HBECs), which showed consistent regulation of SMAD signaling and KIT expression upon *ALK1* knockdown (Figure 5, D–F, and Supplemental Figure 7, B and C), although higher BMP9 concentrations were required to elicit comparable responses (Supplemental Figure 7B).

Moreover, knockdown of *SMAD4* significantly increased KIT levels as well (Figure 5G). These in vitro findings are consistent with recent RNA-seq data showing that brain ECs from *Alk1* and *Smad4* mutant mice exhibit increased *Kit* expression (46) (Supplemental Figure 7, D and E).

In addition to KIT, loss of BMP9/ALK1 signaling also increased the expression of several angiogenic 2 markers in vitro, including *PGF* and *STC1* (Figure 5H and Supplemental Figure 7F). Together, our data indicate that BMP9/ALK1/SMAD4 signaling directly regulates the angiogenic 2 transcriptional program and KIT expression in ECs, rather than acting through secondary changes in cell identity associated with malformation. These findings suggest aberrant KIT signaling may contribute to the initiation of bAVM pathogenesis in *Alk1iECKO* mice.

### Pharmacological KIT inhibition reduces angiogenic reprogramming and vascular malformation in Alk1iECKO mice.

To investigate the functional role of KIT signaling in the initiation of bAVMs, we treated *Alk1iECKO* and control littermate mice with KIT inhibitors (45, 47, 48) or vehicle controls at the time of tamoxifen administration, and we analyzed the PNVP vascular network at P8 (Figure 6A).

Masitinib, a selective RTK inhibitor with its lowest IC_50_ against KIT, significantly reduced vascular area and vessel diameter and prevented vascular malformations induced by ALK1 signaling loss (Supplemental Figure 8, A–C).

Using a KIT-blocking antibody, we observed a marked improvement in PNVP vascular architecture (Figure 6B), accompanied by a significant reduction in vascular area and vessel diameter (Figure 6, C and D). This was associated with a reduction in EC proliferation, as assessed by EdU/ERG colabeling, without evidence of increased apoptosis (Figure 6, E and F, and Supplemental Figure 8D). Despite these vascular improvements, KIT inhibition did not improve the survival of *Alk1iECKO* mice (Figure 6G).

To further investigate the molecular mechanisms underlying these effects, we performed bulk RNA-seq analysis of fresh brain ECs isolated from *Alk1iECKO* and *Alk1l/l* mice treated with KIT-blocking antibody or control antibody (3 mice per condition from 2 independent litters). We first confirmed efficient *Alk1* deletion, as well as reduced expression of canonical downstream targets *Id1* and *Id3* in mutant ECs, in both KIT-blocking antibody– and control-treated conditions (Figure 6H and Supplemental Figure 9A). Consistent with our scRNA-seq findings, we observed an increase in angiogenic 2 cluster markers in *Alk1iECKO* compared with *Alk1l/l* mice, both treated with control antibody (Figure 6H).

Moreover, transcriptomic analyses revealed reductions in KIT signaling–related pathways in *Alk1iECKO* mice treated with KIT-blocking antibody (Supplemental Figure 9B). Importantly, we found a strong modulation of angiogenic 2 cluster–associated genes, such as *Pgf*, *Stc1*, and *Angpt2* (Figure 6H), with only limited changes in control ECs, and RNAscope analysis further validated decreased *Stc1* expression in *Alk1iECKO* PNVP ECs following KIT inhibition (Figure 6I). Broader pathway analysis further supported these trends across hallmark gene sets increased in the angiogenic 2 cluster, such as HYPOXIA, PI3K-AKT-MTOR signaling, and KRAS signaling (Figure 3G and Supplemental Figure 9, C and D).

Next, we leveraged a single-cell drug-repurposing approach (scDrugLink), which integrates gene expression, drug targets, and perturbation signatures to prioritize therapeutic candidates. This analysis revealed that angiogenic 2 ECs displayed high inhibition weights and drug sensitivity scores for several KIT inhibitors, including dasatinib and imatinib (Figure 6J). Given the broad kinase-inhibitory profile of dasatinib, which strongly targets SRC and ABL family kinases in addition to KIT, we prioritized imatinib for in vivo validation to better assess the therapeutic potential of clinically relevant KIT inhibition. Accordingly, mice were treated with imatinib (Gleevec), an FDA-approved multi-kinase inhibitor with activity against KIT. Imatinib treatment improved PNVP vascular architecture (Figure 6, K–M), but it did not significantly affect survival (Figure 6N).

Importantly, neither masitinib, a KIT-blocking antibody, nor imatinib significantly altered the number or diameter of PNVP AV shunts (Supplemental Figure 8C and Supplemental Figure 10, A and B), suggesting that KIT inhibition primarily affects endothelial proliferation and angiogenic reprogramming but is not sufficient to reverse established AV connections under these conditions.

Finally, these drugs had no detectable effect on PNVP vascular architecture, proliferation, apoptosis, or changes in angiogenic 2 marker expression in control mice (Supplemental Figure 8D and Supplemental Figure 10, C–J).

## Discussion

Vascular heterogeneity has emerged as a defining feature of bAVMs, yet the transcriptional programs underlying lesion formation remain poorly understood. Here, we used single-cell transcriptomics to characterize EC heterogeneity and transcriptional reprogramming in a mouse model of HHT2 bAVMs. We found that AVMs form at the brain surface (PNVP) within 48 hours of *Alk1* inactivation, driven by the emergence of a distinct angiogenic EC cluster enriched for *Kit* expression. We further demonstrated that BMP9/ALK1/SMAD4 signaling regulates *Kit* transcription in brain ECs. Finally, we showed that pharmacological inhibition of KIT signaling limits angiogenic reprogramming and vascular malformations in this model.

Our data reveal that the PNVP is more susceptible to AVM formation than the INVP, likely due to its AV architecture and associated hemodynamic forces. This regional vulnerability is consistent with the venous origin of AVMs and their predilection for high-flow regions observed in both mouse models and human brain pathology (39, 49). Trajectory analysis further supports this idea, showing that the angiogenic and proliferative EC clusters driving AVM formation originate from venous ECs. Notably, AVMs emerge in an angiogenic context, yet the PNVP at this developmental stage is undergoing physiological regression, in contrast with the actively expanding INVP (33). It is therefore surprising that loss of ALK1 signaling not only prevents PNVP regression but also induces endothelial proliferation and AVM formation within this normally regressing vascular bed. While the molecular mechanisms governing PNVP regression during postnatal development remain poorly understood, our findings point to ALK1 as a critical regulator of this process.

Moreover, the apparent confinement of AVMs to the PNVP at P8 may reflect a temporal constraint, and in models with longer survival, these malformations are likely to extend into the INVP. In patients, bAVMs are most frequently located in superficial cortical regions but can also occur in deeper structures, including the basal ganglia and thalamus (50). Identifying regional phenotypic differences could lead to the discovery of new molecular drivers involved in bAVM development.

Using a regional PNVP and INVP EC isolation strategy, our study reveals a transcriptional reprogramming of AVM ECs characterized by both proliferative and angiogenic clusters. Genet et al. showed that CDK4/6 inhibitor palbociclib treatment reduces retinal *Alk1iECKO* AVM by limiting EC proliferation (29).

BMP9/ALK1 signaling plays a central role in regulating angiogenic sprouting and tip cell specification (36), and accordingly, *Alk1* deletion in the brain induced the emergence of 2 transcriptionally distinct angiogenic endothelial clusters. Angiogenic 1 ECs, enriched in the INVP, displayed strong transcriptomic similarity to canonical tip cells and appeared to emerge early in the AVM trajectory. RNA velocity analysis suggests that angiogenic 1 ECs may give rise either to sprouting tip cells or to angiogenic 2 ECs associated with AVM formation. The molecular mechanisms governing this fate decision remain unknown but may depend on contextual cues such as hemodynamic forces. As AVMs arise in high-flow environments, elevated shear stress may bias angiogenic 1 ECs toward an angiogenic 2 state, thereby promoting AVM onset.

We identified an angiogenic 2 population characterized by both angiogenic and inflammatory signatures, as previously described by Winkler et al. in human bAVM samples (27). Despite differences between HHT2 mouse models and sporadic human AVMs, this convergence suggests conserved molecular mechanisms. This is particularly striking given the distinct developmental origins and genetic backgrounds involved. Interestingly, established human bAVMs did not exhibit a proliferative EC cluster, suggesting that the angiogenic EC cluster may represent a more relevant target for treating established AVMs.

We found the angiogenic 2 cluster is enriched for *Kit* expression. In mutant ECs, its expression extends beyond tip cells and is enriched in angiogenic populations. Moreover, *SMAD4* knockdown significantly increased KIT levels, consistent with RNA-seq data showing increased *Kit* expression in *Alk1* and *Smad4* mutant brain ECs (46). Therefore, ALK1/SMAD4 signaling negatively regulates *Kit*, and this repression is uncoupled from ENG. These findings suggest that *Kit* upregulation is a feature of ALK1/SMAD4-deficient AVMs but may not be a marker of HHT1-associated lesions.

Importantly, we validated KIT protein expression in human HHT2 and sporadic bAVM endothelium by immunostaining. Together, these findings support the translational relevance of our work and identify KIT as a conserved feature of AVM-associated endothelial reprogramming across species and AVM etiologies, raising the possibility that KIT signaling may represent a shared therapeutic vulnerability across both HHT-associated and sporadic bAVMs.

To prioritize potential therapeutic targets, we applied a single-cell drug-repurposing analysis. This approach revealed that the angiogenic 2 cluster displayed high inhibition weights and sensitivity scores for several KIT-targeting agents such as imatinib and dasatinib. Pharmacological inhibition of KIT signaling reduced endothelial proliferation and angiogenic reprogramming and improved vascular morphology but did not affect survival or established AV shunts, highlighting a stage-specific and partial role of KIT signaling to AVM pathogenesis.

In addition to its well-known function in angiogenesis (51–53), KIT is a potent activator of PI3K/AKT/mTOR and RAS signaling pathways (54), which are central regulators of endothelial metabolism, proliferation, and migration. Consistent with this, we observed a reduction in PI3K/AKT/mTOR and KRAS signaling signatures following KIT inhibition, aligning with the transcriptional programs enriched in angiogenic 2 ECs. KIT signaling also promotes HIF1a expression (55), an important regulator of glycolysis (56, 57), which we found strongly upregulated in angiogenic 2 ECs. KIT may therefore act as an upstream integrator of multiple pathways implicated in AVM pathophysiology, including EC migration, polarity, and flow-responsive signaling (24). Therefore, future studies should evaluate how KIT signaling interacts with other pathways involved in AVM formation.

Finally, KIT signaling inhibition does not rescue survival, indicating a partial functional contribution of this pathway. While bAVMs can lead to severe neurological complications and potentially life-threatening hemorrhage, this dissociation suggests that KIT signaling primarily contributes to brain-specific vascular pathology but is unlikely to underlie the systemic lethality observed in this model. Consistent with this, we did not detect changes in *Kit* expression in lung ECs, where hemorrhagic complications have been proposed to drive mortality.

Overall, this study identifies a bAVM-specific EC population in an HHT mouse model using scRNA-seq and provides a valuable resource for uncovering molecular targets and developing therapeutic strategies for bAVMs.

As for limitations, we identified EC transcriptomic reprogramming at an early stage of bAVM formation, restricting our ability to capture dynamic changes in EC states. A longitudinal analysis at later stages will be critical for defining the ontogeny of AVM EC populations and identifying progressive reprogramming events that could contribute to neurovascular complications. Moreover, although we validated KIT expression in human HHT2 bAVM endothelium, this observation is currently limited by the small number of available samples due to their rarity and will require confirmation in larger patient cohorts.

Finally, although pharmacological inhibition of KIT signaling reduced angiogenic reprogramming and vascular malformations in *Alk1iECKO* mice, we did not assess its efficacy in reversing established AVMs, as treatments were initiated at the onset of lesion formation. Addressing these limitations will be essential for refining our understanding of AVM initiation and progression and for developing targeted therapeutic strategies. In addition, the dosing regimens used in this study were based on preclinical murine models and may not directly translate to human patients. Further dose–response and pharmacokinetic studies will be required to define clinically relevant dosing strategies for bAVMs.

## Methods

### Sex as a biological variable

Both male and female mice were included in this study. Sex was not considered as an experimental variable because all analyses were performed during early postnatal development prior to sexual maturation. Human samples were included based on availability and clinical diagnosis.

### Animal models and experiments

*Alk1* deletion in ECs was achieved by crossing *Alk1l/l* (58) mice with *Cdh5CREERT2* (59) mice to generate *Alk1iECKO* mice. Postnatal deletion was induced via intraperitoneal injection of tamoxifen (Sigma, T5648; 15 μg) at P6. Cre-negative *Alk1l/l* littermates treated with tamoxifen were used as controls. Imatinib mesylate (100 mg/kg, Selleckchem, S1026), masitinib mesylate (100 mg/kg, Cedarlane, E0814), and KIT-blocking antibody (500 μg, InVivoMAb anti-mouse c-Kit, BioXCell, BE0293-5MG-A) were administered intraperitoneally at P6, 3 hours following tamoxifen injection, and again at P7. For imatinib, DMSO served as the control, whereas PBS or an isotype control (500 μg, InVivoMAb rat IgG2b isotype control, BioXCell, BE0090-5MG-A) was used as the control for masitinib and KIT blocking antibody. For the in vivo proliferation assay, mice received an intraperitoneal injection of EdU (30 mg/kg; Life Technologies, A10044) 3 hours before sacrifice. Mice were euthanized at P8, prior to the expected lethality window (~40 hours after tamoxifen), and animals found dead were excluded from all analyses.

For the survival curve, mice were monitored daily for signs of neurological decline, including lethargy, reduced movement, and distress. Humane endpoints were established based on predefined criteria, including excessive weight loss (>20%). Mice that reached predefined endpoints were euthanized in accordance with institutional ethical guidelines.

### Sample fixation and preparation

After euthanasia, brains were fixed in a 4% paraformaldehyde (PFA) solution (Thermo Fisher Scientific, AAJ19943K2) at 4°C overnight.

#### PNVP staining.

The cerebral cortexes were separated, then directly permeabilized and blocked in a blocking buffer containing 0.5% Triton X-100 (Sigma, X100-500ML), 1% BSA (Bioshop, ALB001.100), and 4% donkey serum (Thermo Fisher Scientific, 56-646-05ML) at 4°C overnight. Primary antibodies (Table 1) were added directly to the blocking solution and incubated for 48 hours at 4°C. After 3 PBS washes, secondary antibodies (Table 2) were applied in the blocking solution (1:500 dilution) and incubated for 24 hours at 4°C. Following 3 additional washes, the cortexes were placed on an agarose mold (Figure 1A) and mounted with mounting media (Sigma, 81381-50G). Imaging was performed directly with BioTek Lionheart FX Automated Microscope. Whole-mount preparations correspond to the somatosensory, temporal, and auditory cortical areas.

#### INVP staining.

Brains were immersed in a 30% sucrose (Thermo Fisher Scientific, BP220 1) solution for 48 hours, then embedded and frozen in OCT (Thermo Fisher Scientific, 23-730-571). The immunofluorescence protocol was conducted on 30 μm–thick floating brain sections. Brain sections were permeabilized and blocked overnight in a blocking buffer containing 0.5% Triton X-100, 1% BSA, and 4% donkey serum. Primary antibodies (Table 1) were then incubated in the blocking buffer for 24 hours, followed by PBS washes and a 4-hour incubation with secondary antibodies (Table 2) in the blocking buffer at room temperature. After washing with PBS, brain slices were incubated for 5 minutes with DAPI at a concentration of 2 μg/mL (Sigma, D9542), then mounted using a fluorescent mounting medium. Coronal sections were taken at caudal thalamic levels (approximately bregma –1.46 mm), with imaging focused on the cortical somatosensory area of these sections.

#### Proliferation assay.

At 3 hours before sacrifice mice were intraperitoneally injected with 30 mg/kg of EdU (Life Technologies, A10044, stock solution at 5 mg/mL). The Click-iT EdU cell proliferation kit (Invitrogen, C10340) was used on 100 μm–thick brain sections followed by ERG123 (Abcam, ab92513) immunostaining. Double-positive nucleus staining was counted in order to determine the proportion of the proliferative ECs.

#### ISH.

Floating brain sections were washed 3 times in PBS and mounted on Superfrost Plus microscope slides (Thermo Fisher Scientific, 12-550-15). The slides were air-dried for 10 minutes at room temperature and baked for 30 minutes at 60°C. Samples were fixed in 4% PFA for 15 minutes and dehydrated through a series of ethanol baths with gradually increasing concentrations. After drying, sections were treated with hydrogen peroxide for 10 minutes followed by incubation with Protease IV for 30 minutes. Probe hybridization was carried out at 40°C for 2 hours using RNAscope probes (Table 3). Signal amplification was performed by sequential incubation with RNAscope Multiplex FL v2 Amplification 1 and 2 reagents for 30 minutes each at 40°C, followed by RNAscope Multiplex FL v2 Amplification 3 for 15 minutes at 40°C. Depending on the specific probe, slides were then incubated with the appropriate RNAscope Multiplex FL v2 HRP-C1, HRP-C2, or HRP-C3 reagent for 15 minutes at 40°C. Slides were washed twice for 2 minutes at room temperature using Wash Buffer and incubated with the corresponding fluorophore before the incubation with RNAscope Multiplex FL v2 HRP Blocker for 15 minutes at 40°C. Following final washes, slides underwent immunofluorescence staining for CD31 and were mounted as previously described. Imaging was performed using a Leica TCS SP8 laser scanning confocal microscope.

### Brain preparation for scRNA-seq

Brains from mutant mice and littermate controls (Rep 1 *Alk1l/l* P8 *N* = 2; *Alk1iECKO* P8 *N* = 2, Rep 2 *Alk1l/l* P8 *N* = 2; *Alk1iECKO* P8 *N* = 2) were collected and directly immersed and digested using the Neural Tissue Dissociation Kit (P) (Miltenyi Biotec, 130-092-628). Each brain was digested at 37°C for 30 minutes. During the enzymatic reaction, gentle flushing of the brain tissue was performed to separate the PNVP from the rest of the brain. The reaction was stopped by adding DMEM containing 5% FBS, and the enzymatic solution containing the PNVP was filtered through a 40 μm cell strainer (Falcon, 352340). The remaining brain tissue was rinsed with PBS, mechanically dissociated, and subjected to a second round of enzymatic digestion. After further homogenization by pipetting, digestion was stopped with 5% FBS in DMEM, and the suspension was again passed through a 40 μm cell strainer. Myelin and cell debris were removed using the Debris Removal Solution (Miltenyi Biotec, 130-109-398), which separates components into distinct phases. Red blood cells were lysed with Red Blood Cell Lysis Solution (Miltenyi Biotec, 130-094-183). The resulting cell suspension was washed with a FACS buffer (PBS containing 1% FBS and 0.2% EDTA). Cells were stained for 30 minutes at room temperature with PE anti-mouse CD140b Antibody (BioLegend, 136006), APC-conjugated anti-mouse CD31 antibody (BD Biosciences, 551262), and FITC-conjugated anti-mouse CD45 antibody (BioLegend, 103108) in Fc block solution (5 μg/mL CD16/CD32 Fc Block, eBioscience, 16-0161-82). After washing, cells were stained with Live/Dead Fixable Aqua Dead Cell Stain (Thermo Fisher Scientific, L34957) for 20 minutes at room temperature. Stained cells were washed and resuspended in PBS. The gating strategy was defined using unstained and single-stained controls. Dead cells and doublets were excluded, and ECs (CD45^–^CD31^+^) were sorted using a BD FACSAria Fusion. Cells were resuspended in PBS containing 0.04% BSA and subjected to scRNA-seq.

Four libraries (*Alk1l/l* PNVP, *Alk1l/l* INVP, *Alk1iECKO* PNVP, *Alk1iECKO* INVP) were generated using the Chromium Single Cell 3’ Reagent Kits v3.1 for each experiment (10x Genomics). Libraries were sequenced on Illumina NovaSeq6000 S4 (mean of ~144,000 reads/cell), followed by demultiplexing and mapping to the mouse genome (Mus_musculus:GRCm38) using Cell Ranger (6.1.1, 10x Genomics). Count matrices were analyzed using Seurat V5 (60). Doublets were identified using the Solo package (61, 62) and removed from further analysis. Genes expressed in fewer than 10 cells were excluded. We kept cells with more than 200 genes and fewer than 6,500 genes, fewer than 45,000 transcripts, and less than 10% mitochondrial transcripts. Following quality control and initial dimensionality reduction, ECs were then subclustered and analyzed separately. Briefly, UMAP-identified ECs within each of the 4 datasets for each experiment (Rep 1 *Alk1l/l* PNVP = 1,857 ECs, *Alk1l/l* INVP = 3,215 ECs, *Alk1iECKO* PNVP = 2,193 ECs, *Alk1iECKO* INVP = 1,857 ECs) were merged together (mean nFeature/cell = 4,097, mean nCount/cell = 16,763) and normalized using SCTranform (regression on nFeature_RNA and percent.mt), and then dimensionality reduction was performed using runUMAP based on the first 20 PCs from principal component analysis of the top 3,000 most variable genes (SCTransform assay). Clustering was then performed on UMAP first and second dimensions at a resolution of 1.2, followed by differential marker expression analysis between clusters based on normalized RNA count (RNA assay). Normalized RNA counts were then scaled (regression on nFeature_RNA and percent.mt) for data visualization. Clusters were finally annotated based on known endothelial subtype marker genes. Gene expression from the RNA assay was visualized using featureplot or dotplot functions. Pathway activity score computation using the MsigDB database (msigdb.v2023.1.Mm.symbols.gmt) or in-house datasets was performed based on gene counts from the SCT assay using the package VISION (https://doi.org/10.1038/s41467-019-12235-0). Differential gene expression or pathway score was done using the Wilcoxon rank sum test and visualized on a log2 fold-change basis. Trajectory analysis was performed with Monocle3 package (https://cole-trapnell-lab.github.io/monocle3/) using normalized RNA counts and precomputed UMAP dimensions from Seurat analysis. Pseudotime analysis was performed on Monocle3 graph trajectory using the vein subtype as root.

Cell-cell communication (CCC) analysis was performed using the Connectome package (https://msraredon.github.io/Connectome/) on the RNA assay, computing a *z*-score for each ligand-receptor pair between cell types. CCC was visualized using Circos plot using thresholds on percentage of ligand and receptor expression in each cell type as well as ligand-receptor *z*-score. Public datasets from Winkler et al. and Wälchli (27, 28) et al. were downloaded (https://adult-brain-vasc.cells.ucsc.edu and https://brain-vasc.cells.ucsc.edu) as a preannotated Seurat object and used directly for gene expression and/or Connectome analysis as described above.

We applied the scDrugLink method to predict drug sensitivity and inhibition effects across EC subclusters. This approach integrates drug target expression with transcriptomic reversal scores derived from CMAP L1000 perturbation signatures to compute drug-specific scores for each cell type. Mutant and control choroid EC clusters were excluded from the analysis to focus on brain-relevant populations. The angiogenic 2 cluster was compared with all EC clusters from *Alk1l/l* control mice. Drug sensitivity/resistance and inhibition/promotion scores were calculated and aggregated using the scDrugLink R package (https://github.com/LHBCB/scDrugLink; commit ID 31787cc). For each drug, the inhibition weight in a given cell type was computed by integrating Cliff’s Delta and the adjusted *P* value derived from a within-cell-type Wilcoxon rank-sum test comparing mutant and control cells. Drugs with both high inhibition weights and high sensitivity scores were prioritized as repurposing candidates for targeting angiogenic 2 ECs.

We replicated our scRNA-seq (Rep 2 *Alk1l/l* PNVP = 2,179 ECs, *Alk1l/l* INVP = 5,331 ECs, *Alk1iECKO* PNVP = 3,044 ECs, *Alk1iECKO* INVP = 6,551 ECs) and integrated replicate 2 with replicate 1 using Seurat IntegrateLayers functions on SCT assay, followed by dimensionality reduction as described above. Subtype of ECs making less than 1% of total ECs per condition were removed.

### Human immunofluorescence staining

Human brain tissue specimens and associated clinical data were obtained at the University of California, San Francisco, under protocols approved by the institutional review board and ethics committee. Tissue specimens were collected from patients undergoing neurosurgical procedures after written informed consent for research use. To minimize artifactual tissue disruption, specimens were harvested in close coordination with neurosurgeons trained in tissue isolation. Specimens were fixed in formalin and embedded in paraffin using standard protocols. Formalin-fixed, paraffin-embedded sections were cut at 6 μm, deparaffinized in xylene, and rehydrated through graded ethanol washes to distilled water. Antigen retrieval was performed in pH 9 target retrieval solution (Agilent, GV80411-5) at 85°C for 15 minutes. Sections were then blocked for 1 hour at room temperature in PBS containing 10% donkey serum, 0.05% gelatin, and 0.1% Triton, followed by incubation with primary antibodies overnight at 4°C. Primary antibodies included podocalyxin (1:100, R&D Systems, AF1658-SP) and CD117/c-KIT (1:100, Agilent, A450229-2). After washing in PBS containing 0.05% Triton, sections were incubated for 2 hours at room temperature, protected from light, with fluorophore-conjugated secondary antibodies: Alexa Fluor 555 donkey anti-rabbit (1:500, Thermo Fisher Scientific, PIA32794) and Alexa Fluor 647 donkey anti-goat (1:500, Thermo Fisher Scientific, PIA32849). Sections were then washed and treated with 1% Sudan Black (Thermo Fisher Scientific, BP109-10) for 10 minutes at room temperature to quench autofluorescence. Nuclei were counterstained with DAPI for 30 minutes at room temperature. Slides were washed in PBS and mounted with antifade mounting medium (Vector Laboratories, H-1700-10). Images were acquired on an Eclipse Ti2 microscope (Nikon Instruments).

### HUVECs and HBECs’ culture

HUVECs (Promo Cell, Cedarlane, C-12203) and HBECs (Cell Systems, ACBRI 376) were cultured in EBM2 Basal medium (Lonza CC-3121) supplemented with the SingleQuots Supplements (Lonza CC-4133). Cells were transfected with different siRNAs (Table 4) using Lipofectamine RNAiMAX (Invitrogen, 13778150). At 48 hours after transfection, cells were treated with BMP9 (R&D Systems, 3209-BP-010) at 5 ng/mL or 20 ng/mL during 24 hours.

### mBECs’ isolation and bulk RNA sequencing

Mutant mice and littermate controls were injected with a KIT-blocking antibody (500 μg, InVivoMAb anti-mouse c-Kit) or an isotype control (500 μg, InVivoMAb rat IgG2b isotype control) at P6 and P7. CD31 antibody (BD Pharmingen, 550274) was incubated with Dynabeads (Thermo Fisher Scientific, 11035) at 4°C overnight. Beads were then washed with PBS containing 0.1% BSA using a magnetic separator (DynaMag-15, Thermo Fisher Scientific, 12301D) and resuspended in PBS containing 0.01% BSA. At P8, mice were euthanized, and brains were collected, finely chopped, and digested for 1 hour at 37°C in Collagenase/Dispase (Sigma, 11097113001) supplemented with DNase I (Sigma DN25) and TLCK (0.147 μg/mL, tosyl-lysine-chloromethylketone, Sigma T7254). The reaction was stopped with DMEM containing 5% FBS, and the suspension was filtered through a 40 μm cell strainer (Thermo Fisher Scientific, 087711). Myelin and debris were removed using a 25% BSA gradient in DMEM. After a wash with PBS, cells were incubated in PBS containing 0.1% BSA with 100 μL of CD31-coated beads for 30 minutes at room temperature under agitation. Cells were then washed using a magnetic separator and lysed, and total RNA was extracted using the Monarch Total RNA Miniprep Kit (New England Biolabs [NEB], T2010S). Following quality control, RNA libraries were prepared using a poly(A)-enriched RNA library preparation protocol and sequenced in 2 batches on an Illumina NovaSeq platform (paired-end 100 bp, ~25 million reads per sample ± 5 million reads; Genome Québec). Raw sequencing data were processed using the DRAGEN RNA pipeline (Illumina) to generate gene count tables. Further analysis was performed using the limma-voom package in R (63). The DRAGEN quant.genes.sf file was annotated with the gencode.vM38.basic.annotation.gtf file to obtain gene-level counts from transcript-level data. Differential expression analysis was conducted using the edgeR package (64). Count data were filtered and normalized using the calcNormFactors function with the trimmed mean of M-values method. Data were log_2_-transformed, and multidimensional scaling (MDS) analysis was performed using the plotMDS function to visualize sample distributions in a 2D scatterplot. Differential gene expression analysis was carried out using the limma-voom pipeline, including the voom, lmFit, contrasts.fit, eBayes, and decide Tests functions. Batch correction was performed by design in limma-voom function.

### mLEC isolation for cell culture

Mouse lung epithelieal cell CD31 antibody (BD Pharmingen, 550274) was incubated with Dynabeads (Thermo Fisher Scientific, 11035) at 4°C overnight and then washed with PBS-0.1%BSA using magnetic separator DynaMag-15 (Thermo Fisher Scientific, 12301D) and resuspended in PBS-BSA 0.01%. Lungs were digested 1 hour at 37°C using Collagenase type I (Worthington, LS004196). Reaction was stopped with DMEM 5% SVF, filtered through a 70 μm cell strainer (Thermo Fisher Scientific, 087712). After 1 step of washing, the pellet was resuspended in PBS-0.1% BSA, and 100 μL of beads coated with CD31 were added and incubated 30 minutes, at room temperature, under agitation. Using the magnetic separator, cells were washed with PBS and then subjected to cell lysis.

### mBECs culture

For cell culture PNVP was separated enzymatically from INVP as previously described. Myelin and debris were removed with DMEM-BSA 25% gradient, and after 1 step of wash with PBS, cells were seeded in EBM2 Basal medium with SingleQuots Supplements (Lonza CC-4133) and 20% FBS (Thermo Fisher Scientific, 12484028) on plates coated with collagen I (Thermo Fisher Scientific, A1064401). Selection of ECs was made with puromycin (Santa Cruz Biotechnology, SC-108071A) at 10 μg/mL for 2 days and after at 2 μg/mL.

For RNA extraction, total brain was digested with Collagenase/Dispase (Sigma, 11097113001) for 1 hour supplemented with DNAse I and TLCK. Myelin and cell debris were removed as previously described, and ECs were isolated using beads coated with CD31 and the magnetic separators.

### Quantitative real-time PCR

Total RNA was extracted using Monarch Total RNA Miniprep Kit (NEB, T2010S), reverse transcription used iScript Reverse Transcription Supermix (Bio-Rad, 1708841), and RT-qPCR used iQ SYBR Green Supermix (Bio-Rad, 1708882) and primers (Table 5) Data were normalized with housekeeping genes, and mRNA relative expression was calculated using the double-CT method.

### Western blot

HUVECs or HBECs were lysed with Laemmli’s buffer 4× (Bio-Rad, 1610747), and protein lysates were loaded in NuPAGE MES SDS Running Buffer 20× (Thermo Fisher Scientific, NP0002), transferred on nitrocellulose membrane (Bio-Rad, 1620112), and blocked 1 hour at room temperature on 3% BSA-TBS with Tween (TBST) before the incubation with primary antibody (Table 1) at 4°C overnight. The day after, membranes were washed with TBST and incubated 1 hour at room temperature horseradish peroxidase–labeled secondary antibodies (Table 2), and signal was revealed using SuperSignal West Femto Maximum Sensitivity Substrate (Thermo Fisher Scientific, 34096) or Clarity Western ECL substrate (Bio-Rad, 1705060S) on ChemiDoc MP imaging system (Bio-Rad). Analysis and quantification were made using Image Lab software (Bio-Rad).

### Statistics

Statistical analysis and graph images were generated using GraphPad Prism 10 software. Comparisons across multiple groups were conducted using ordinary 1-way ANOVA (for normally distributed data with equal variances), Welch’s ANOVA (for normally distributed data with unequal variances), or the nonparametric Kruskal-Wallis test (for non-normally distributed data). Statistical comparisons between 2 groups were conducted using an unpaired *t* test (for normally distributed data with equal variances), Welch’s *t* test (for normally distributed data with unequal variances), or the Mann-Whitney *U* test (for non-normally distributed data), as appropriate. All *t* tests were 2-tailed. Survival curves were analyzed using the Kaplan-Meier method and compared with the log-rank test. *P* < 0.05 was considered statistically significant. All data are represented as mean ± SEM.

### Study approval

All breeding colonies and experimental protocols were approved by the Institutional Committee on Animal Research Ethics (CIBPAR). Human brain tissue collection and analysis were approved by the Institutional Review Board of the University of California, San Francisco, and written informed consent was obtained from all participants or their legal representatives.

### Data availability

Materials are available upon reasonable request.

All data that support the findings of this study are available within the article and its supplemental files and from the corresponding author upon reasonable request.

The RNA-seq and scRNA-seq data discussed herein are deposited in NCBI’s Gene Expression Omnibus (accession no. GSE328234 and GSE297553, respectively). Code is available on GitHub (https://github.com/gaelcge/brainAVM_Drape_scRNAseq; commit ID 179609d).

Supporting Data Values are provided in a separate XLS file.

## Author contributions

ED, BL, and AD conceptualized and designed the study. ED, MB, and LC conducted histological experiments; ED and PPVV performed single-cell transcriptomic and RNA-seq experiments; and GC, JPH, and TA analyzed the bioinformatic data. ED, LC, ARMF, HDTW, MB, JW, and MI analyzed the data. DS, AG, and EW performed histological analyses on human AVM samples. AD secured funding for the project. ED and AD prepared the initial draft of the manuscript. GA, along with all authors, contributed to reviewing and editing the final text.

## Conflict of interest

The authors have declared that no conflict of interest exists.

## Funding support

- Project grants from the Canadian Institutes of Health Research (2019PJT-165871, 202203PJT-183658, 202403PJT-517269 to AD).
- The Natural Sciences and Engineering Research Council of Canada (RGPIN-2022-04726 to AD).
- ED by fellowships from Vision Science Research Network (RRSV) and CHU Sainte-Justine Foundation.
- LC by a fellowship from CHU Sainte-Justine Foundation.
- ED and LC were supported by salary awards from the Office of Graduate and Postdoctoral Studies (Études supérieures et postdoctorales, ESP), at the University of Montréal.

## Supplementary Material

Unedited blot and gel images

Supplemental table 1

Supporting data values

## Figures and Tables

**Figure 1:**
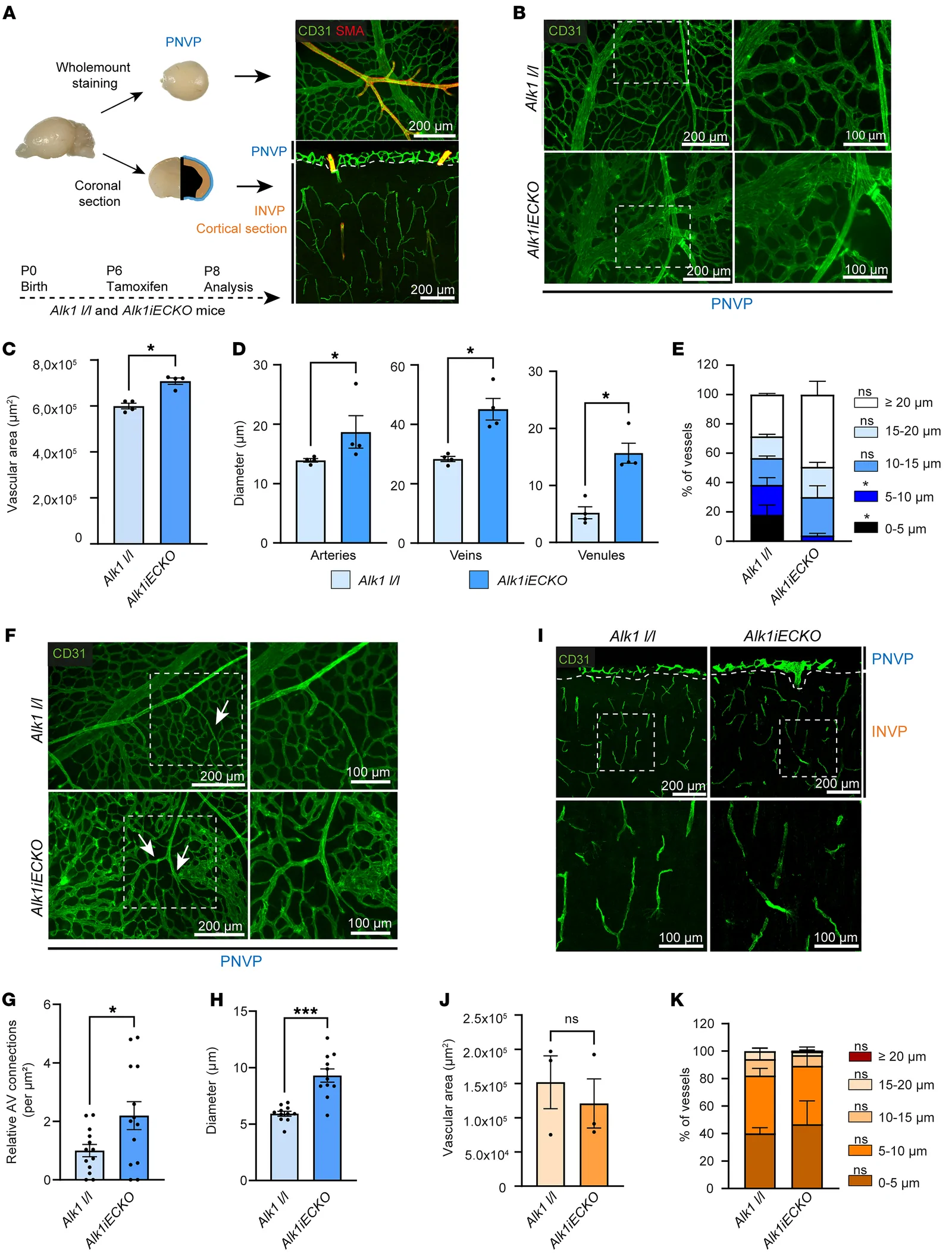
Loss of ALK1 signaling induces vascular malformations in the PNVP. (**A**) Left, experimental timeline of tamoxifen administration and tissue analysis in *Alk1iECKO* and control mice. Right, representative images showing CD31 and α-SMA immunostaining of the PNVP and INVP at P8, indicating vascular morphology. α-SMA, α–smooth muscle actin; PNVP, perineural vascular plexus; INVP, intraneural vascular plexus; *l/l*, lox/lox. (**B**) CD31 immunostaining of the PNVP of *Alk1iECKO* and control mice at P8. (**C**) Quantification of vascular area in the PNVP of *Alk1iECKO* and control mice (*n* = 4 for each condition). (**D** and **E**) Quantification of vessel diameter in the PNVP of *Alk1iECKO* and control mice at P8 (*n* = 4 for each condition). (**F**) CD31 immunostaining showing direct AV connections in the PNVP of *Alk1iECKO* mice. (**G**) Quantification of PNVP AV shunts in the PNVP of *Alk1iECKO* and control mice (*n* = 13 for each condition). (**H**) Quantification of PNVP AV shunt diameter in the PNVP of *Alk1iECKO* and control mice at P8 (*n* = 11 for each condition). (**I**) CD31 immunostaining of brain sections from *Alk1iECKO* and control mice at P8. (**J** and **K**) Quantification of vascular area (*n* = 3 for each condition) (**J**) and vessel diameter (*n* = 3 for each condition) (**K**) in the INVP of *Alk1iECKO* and control mice at P8. Each dot represents 1 mouse. Data represent means ± SEM. *P < 0.05, ***P < 0.001, Mann-Whitney test (**C**–**E**, **G**, **H**, and **K**), Welch’s *t* test (**D**, **E**, **G**, and **H**), or unpaired *t* test (**E**, **J**, and **K**) was performed.

**Figure 2:**
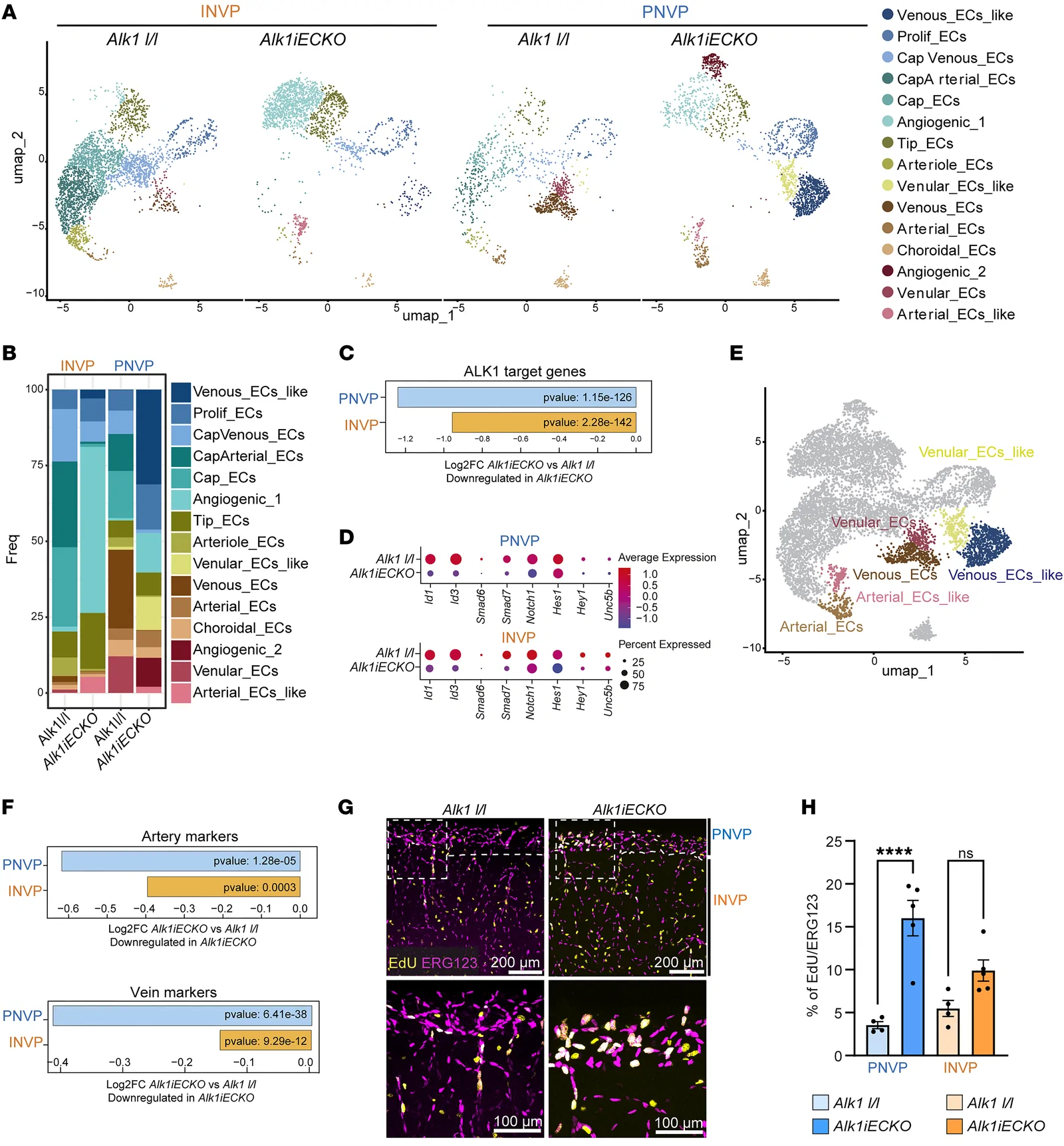
ScRNA-seq reveals distinct EC reprogramming in *Alk1iECKO* brains. (**A**) UMAP plots of EC clusters from INVP and PNVP of *Alk1iECKO* and control brains obtained by scRNA-seq. (**B**) Proportion of EC clusters in indicated conditions. (**C**) Bar plot of VISION score log2FC (*Alk1iECKO* vs. control) of ALK1 signaling target gene set in PNVP and INVP ECs. (**D**) Dot plot of the ALK1 signaling target genes’ expression. (**E**) UMAP plot of EC clusters from INVP and PNVP of *Alk1iECKO* and control brains highlighting arterial, venular, and venous ECs. (**F**) Bar plot of VISION score log2FC (*Alk1iECKO* vs. control) of arterial (top) and venous (bottom) gene set signatures in PNVP and INVP ECs. (**G**) Double ERG and EdU immunolabeling of P8 *Alk1iECKO* and control brains. The white dotted line delineates the boundary between the PNVP and the INVP. The white dotted box indicates the region shown at higher magnification below. (**H**) Quantification of ERG^+^EdU^+^/ERG^+^ cells in PNVP and INVP from *Alk1iECKO* (*n* = 5) and control (*n* = 4) mice. Each dot represents 1 mouse. Data represent means ± SEM. ****P < 0.0001. Ordinary 1-way ANOVA (**H**) and Wilcoxon’s rank sum test (**C** and **F**) were performed.

**Figure 3:**
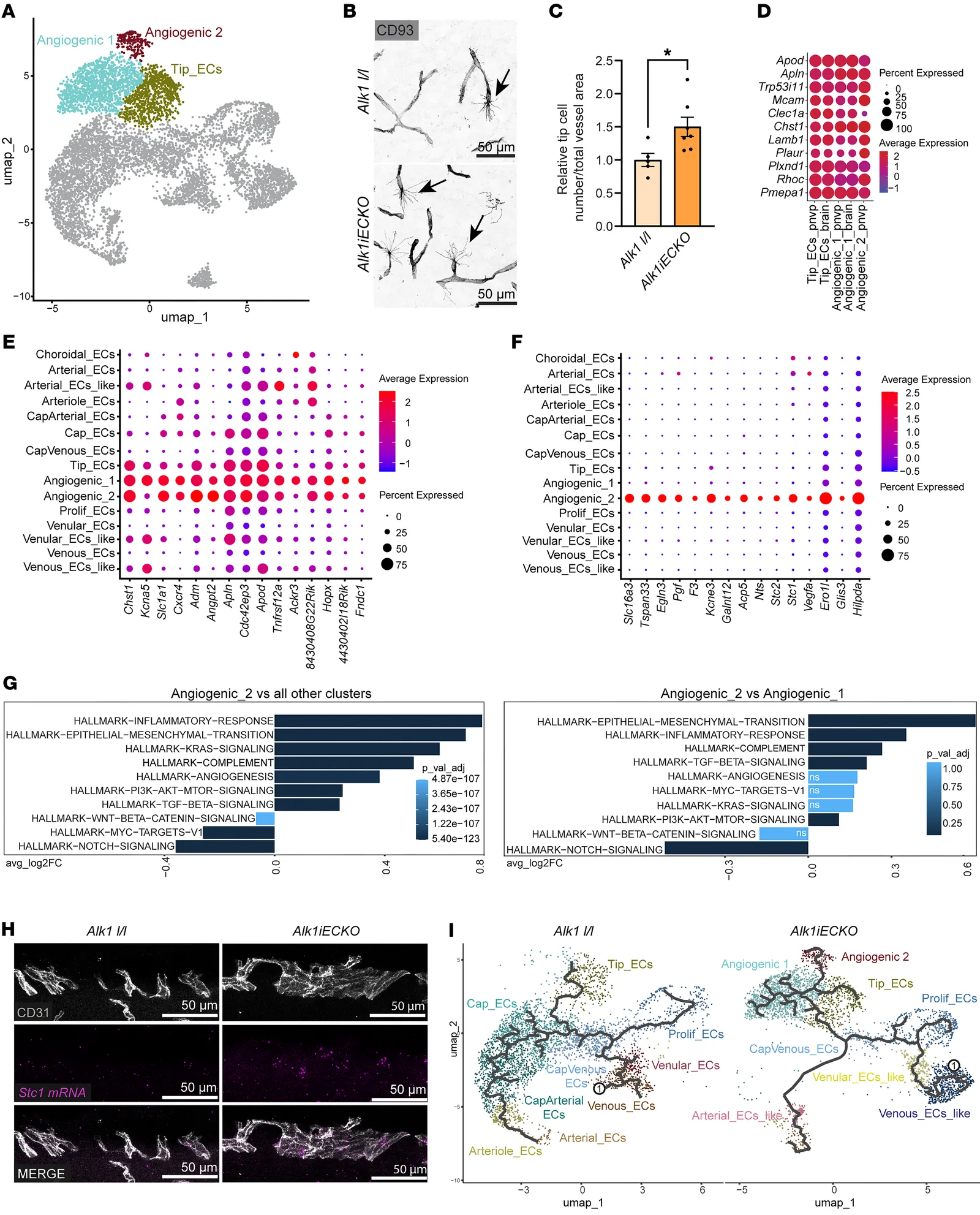
Characterization of PNVP-enriched angiogenic EC clusters in *Alk1iECKO* brains. (**A**) UMAP plots of EC clusters from INVP and PNVP of *Alk1iECKO* and control brains highlighting angiogenic clusters. (**B**) CD93 immunostaining showing an increase in tip cell number in the INVP of *Alk1iECKO* mice. (**C**) Quantification of tip cell numbers in *Alk1iECKO* (*n* = 7) and control (*n* = 5) INVP. Each dot represents 1 mouse. (**D**) Dot plot of tip cell markers’ expression. (**E** and **F**) Dot plot of top 15 angiogenic 1 (**E**) and angiogenic 2 (**F**) cluster markers. (**G**) Bar plot of VISION score log2FC in angiogenic 2 ECs against all other clusters for the top 10 most significant pathways from the hallmark MSigDB gene sets. (**H**) ISH for *Stc1* mRNA confirming increased expression in *Alk1iECKO* PNVP ECs. (**I**) Trajectory analysis of ECs from control and *Alk1iECKO* mice at P8. Data represent means ± SEM. *P < 0.05. Mann-Whitney test (**C**) and Wilcoxon’s rank sum test (**G**) were performed.

**Figure 4:**
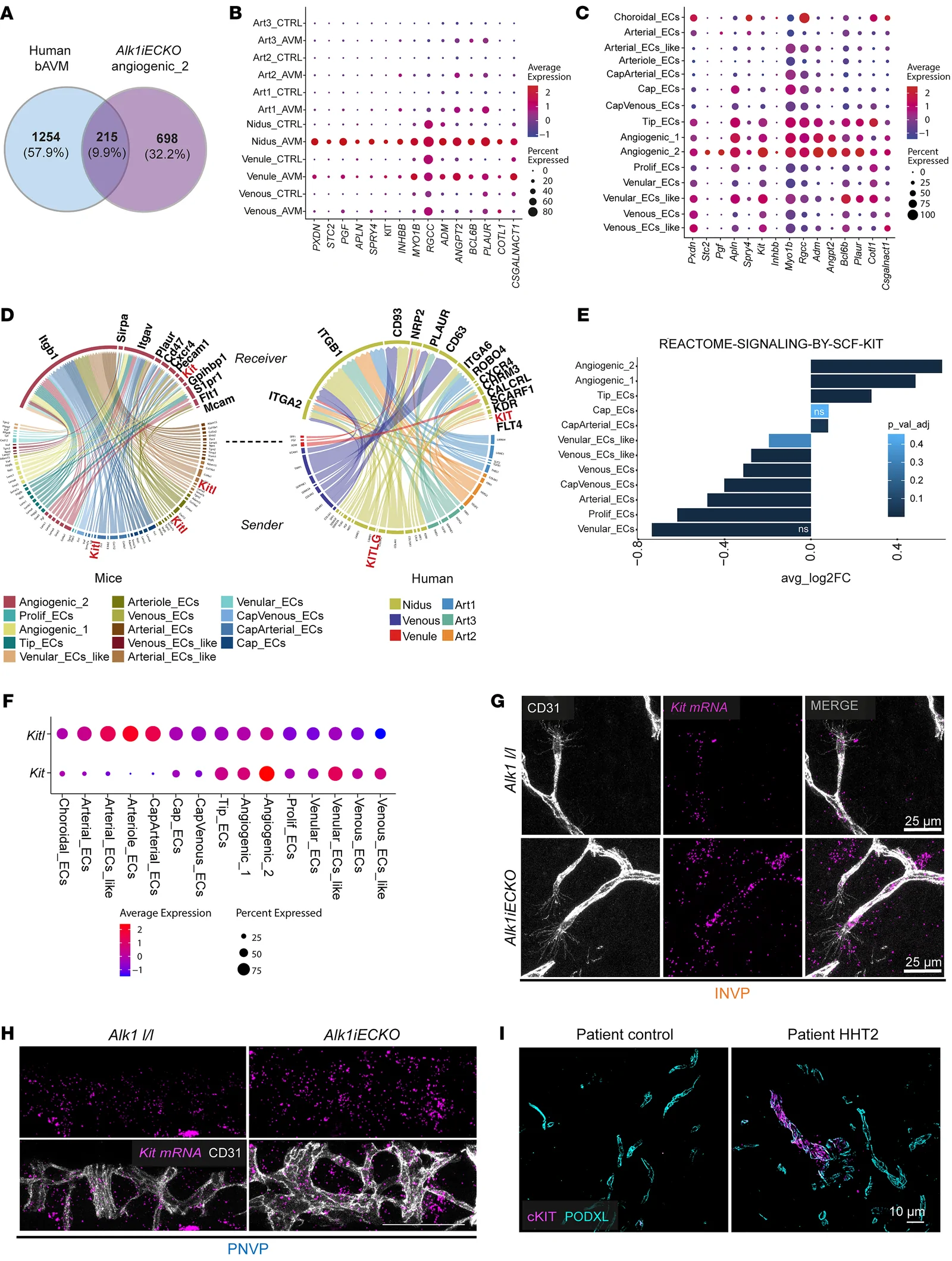
Mouse *Alk1iECKO* angiogenic 2 and human AVM endothelial clusters share AVM molecular markers, including KIT. (**A**) Venn diagram of marker genes (log2FC ≥ 0.25, adjusted *P* value ≤ 0.05) from human brain nidus and *Alk1iECKO* angiogenic 2 cluster. (**B** and **C**) Dot plot of top 10 common markers’ expression in human (**B**) and mouse (**C**) ECs. (**D**) Circos plot representation of enriched ligand-receptor interactions in mouse (min.pct 0.5, min.z 0.5) and human (min.pct 0.2, min.z 0.5) EC clusters. (**E**) Bar plot of VISION score log2FC of KIT signaling between mouse EC clusters. (**F**) Dot plot of *Kit* and *Kitl* expression. (**G**) ISH for *Kit* mRNA using brain coronal sections of P8 *Alk1iECKO* and control mice. (**H**) ISH for *Kit* mRNA and CD31 immunostaining of PNVP ECs. (**I**) Double KIT and podocalyxin (PODXL) immunostaining of brain vessels from control and HHT2 patients. Wilcoxon’s rank sum test (**A** and **D**–**F**) was performed.

**Figure 5:**
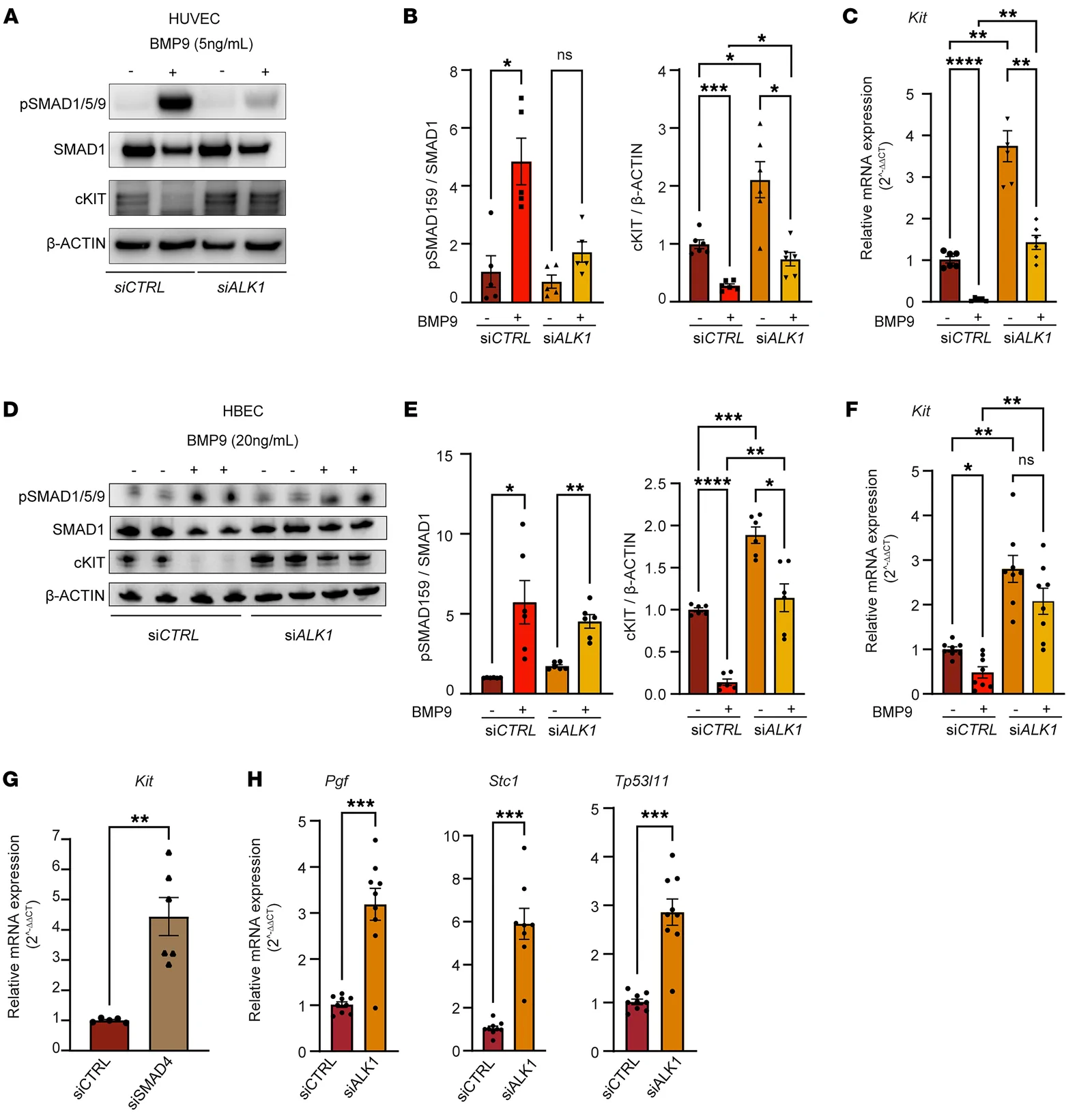
ALK1 signaling suppresses *KIT* and angiogenic 2 markers’ expression in vitro. (**A**) Immunoblot detection of p-SMAD1/5/9, total SMAD1, KIT, and β-actin in HUVECs treated or not with BMP9 (5 ng/mL, 24 hours) and transfected with control or *ALK1* siRNA. (**B**) Quantification of p-SMAD1/5/9/SMAD1 (left) and KIT/β-actin (right) protein levels of immunoblots corresponding to **A** (*n* = 6). (**C**) RT-qPCR analysis of *KIT* expression in HUVECs treated with *ALK1* siRNA compared with control (*n* = 6 derived from 3 independent experiments). (**D**) Immunoblot analysis of p-SMAD1/5/9, total SMAD1, KIT, and β-actin in HBECs transfected with control or *ALK1* siRNA. (**E**) Quantification of p-SMAD1/5/9/SMAD1 (left) and KIT/β-actin (right) corresponding to **D** (*n* = 6 derived from 3 independent experiments). (**F**) RT-qPCR analysis of *KIT* expression in HBECs following *ALK1* knockdown (*n* = 8 derived from 4 independent experiments). (**G**) RT-qPCR analysis of *KIT* expression in HUVECs treated with *SMAD4* siRNA compared with control (*n* = 6 derived from 2 independent experiments). (**H**) RT-qPCR analysis of selected angiogenic 2 markers in HUVECs treated with *ALK1* siRNA compared with control (*n* = 9 derived from 5 independent experiments). Each dot represents 1 experiment. Data represent means ± SEM. *P < 0.05, **P < 0.01, ***P < 0.001, ****P < 0.0001. Brown-Forsythe and Welch ANOVA (**B**, **C**, **E**, and **F**) and Welch’s *t* test (**G** and **H**) were performed.

**Figure 6:**
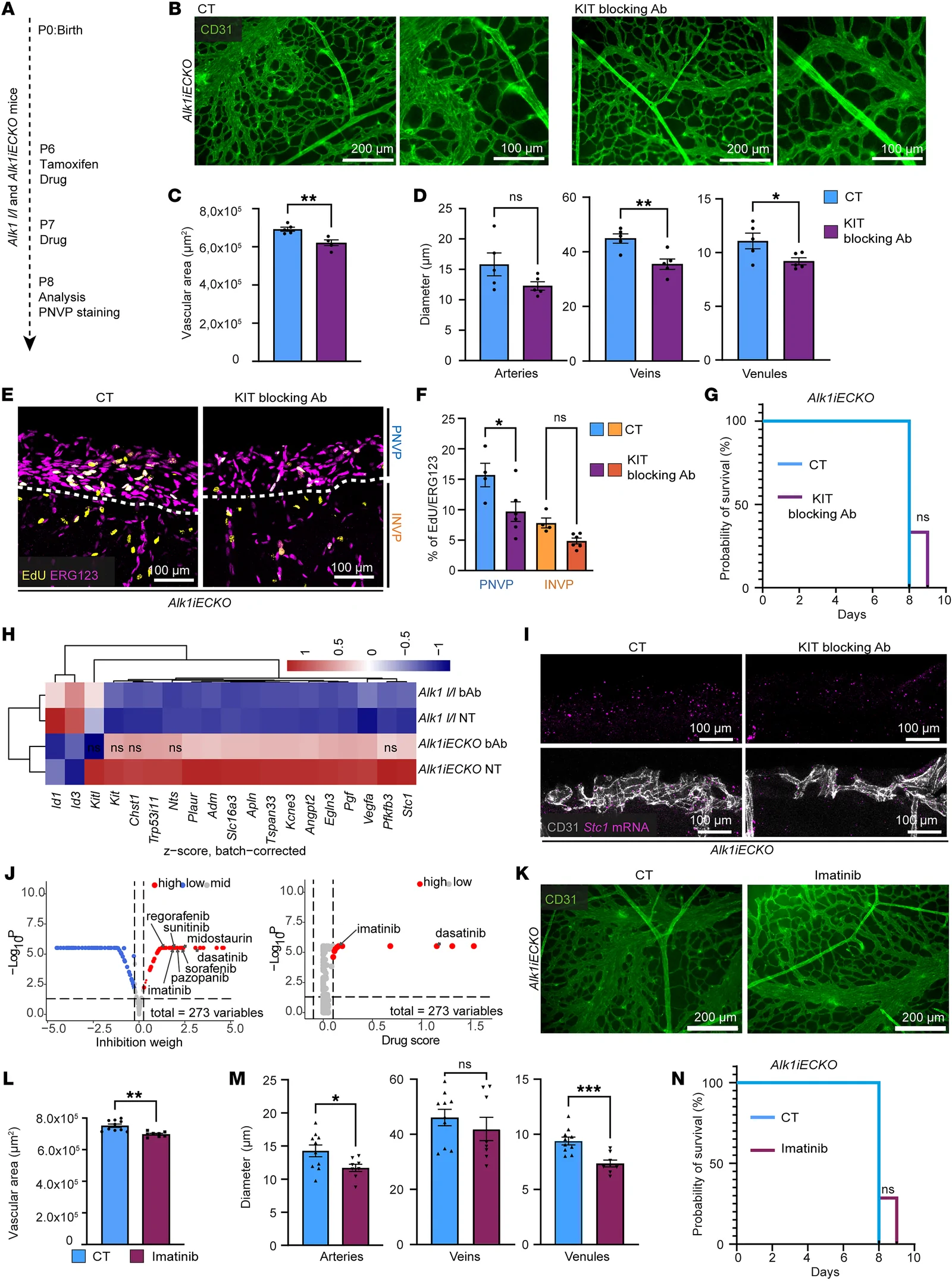
Pharmacological KIT inhibition prevents EC proliferation and brain vascular malformations in *Alk1iECKO* mice. (**A**) Experimental timeline of tamoxifen and KIT inhibitor administration in *Alk1iECKO* and control mice. (**B**) CD31 immunostaining of the PNVP of P8 *Alk1iECKO* mice injected with KIT-blocking antibody or control (CT). (**C** and **D**) Vascular area (**C**) and vessel diameter (**D**) quantifications in the PNVP of P8 *Alk1iECKO* mice (*n* = 5). (**E**) EdU incorporation and ERG123 immunostaining in the PNVP of control and KIT-blocking antibody–treated *Alk1iECKO* mice. (**F**) EdU^+^/ERG^+^ EC quantification in control (*n* = 4) and KIT-blocking antibody– (*n* = 6) treated *Alk1iECKO* mice. (**G**) Survival curve of *Alk1iECKO* mice treated with KIT-blocking antibody (*n* = 6) or control (*n* = 4). (**H**) Heatmap showing scaled gene expression of angiogenic markers assessed by RNA-seq in mouse brain endothelial cells (mBECs) from *Alk1iECKO* and control mice treated with KIT-blocking antibody or control (*n* = 3). (**I**) RNAscope detection of *Stc1* mRNA with CD31 immunostaining in the PNVP of *Alk1iECKO* mice treated with KIT-blocking antibody or control. (**J**) Drug-repurposing analysis applied to the *Alk1iECKO* angiogenic 2 EC cluster. Left: Network-based inhibition weight of angiogenic 2 cluster compared with all control EC clusters. Right: Top candidate drugs ranked by predicted drug score. (**K**) CD31 immunostaining of the PNVP of P8 *Alk1iECKO* mice injected with imatinib or control. (**L**) Vascular area quantification in the PNVP of P8 *Alk1iECKO* mice treated with imatinib (*n* = 8) or control (*n* = 10). (**M**) Vessel diameter quantification in the PNVP of P8 *Alk1iECKO* mice treated with imatinib (*n* = 8) or control (*n* = 10). (**N**) Survival curve of *Alk1iECKO* mice treated with imatinib or control. Data represent means ± SEM. *P < 0.05, **P < 0.01, ***P < 0.001. Unpaired *t* test (**C**, **D**, **L**, and **M**), Welch’s *t* test (**M**), ordinary 1-way ANOVA (**F**), and limma-voom moderated *t* test (**H**) were performed. Each dot represents 1 mouse. For survival curves Kaplan-Meier methods were performed, using log-rank test. For the heatmap batch-corrected limma-voom test was performed, and non-significant values correspond to comparison between *Alk1iECKO* KIT-blocking antibody vs. CT mice.

**Table 5:**
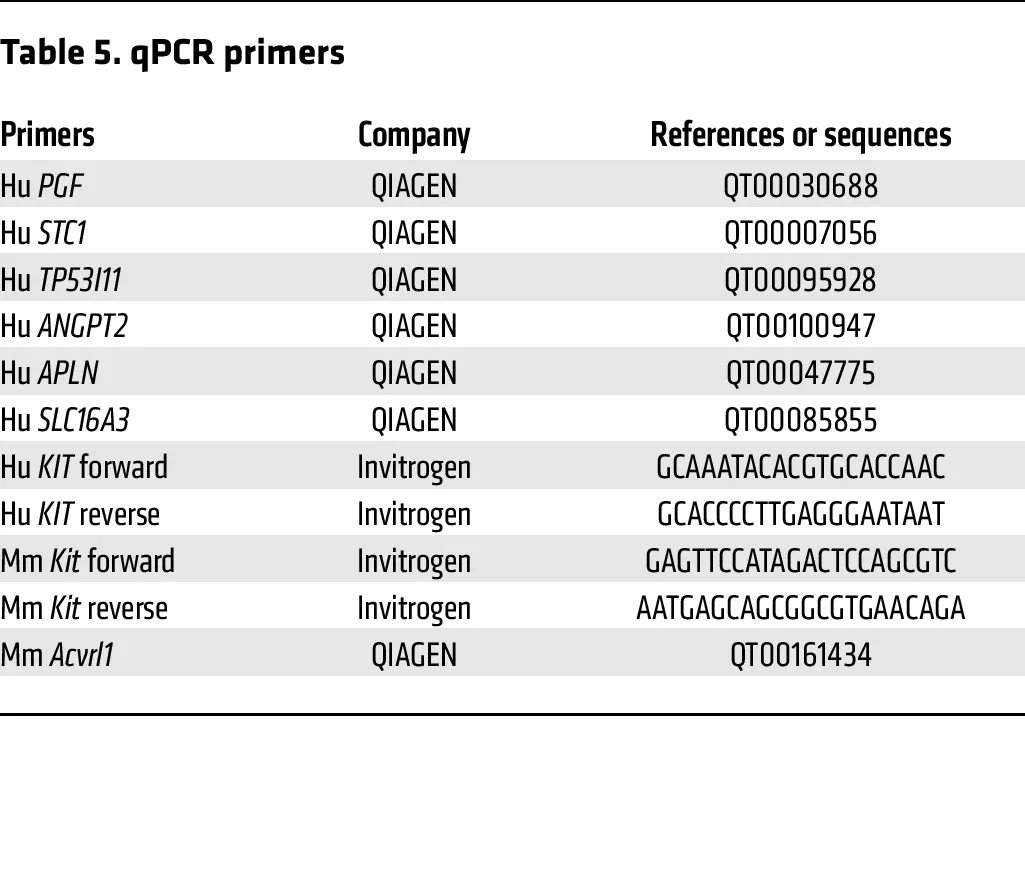
qPCR primers

**Table 4:**
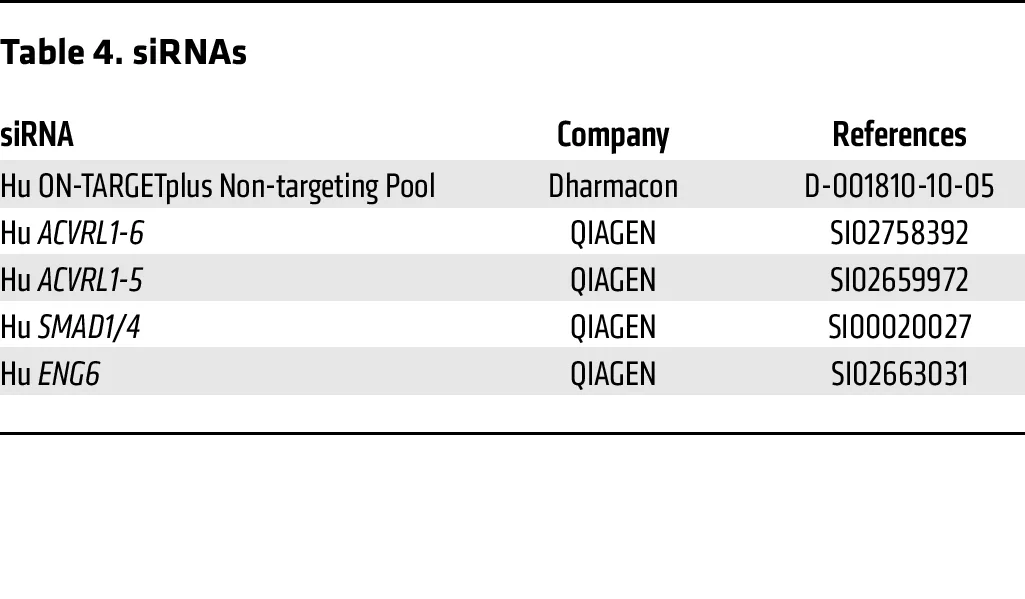
siRNAs

**Table 3:**
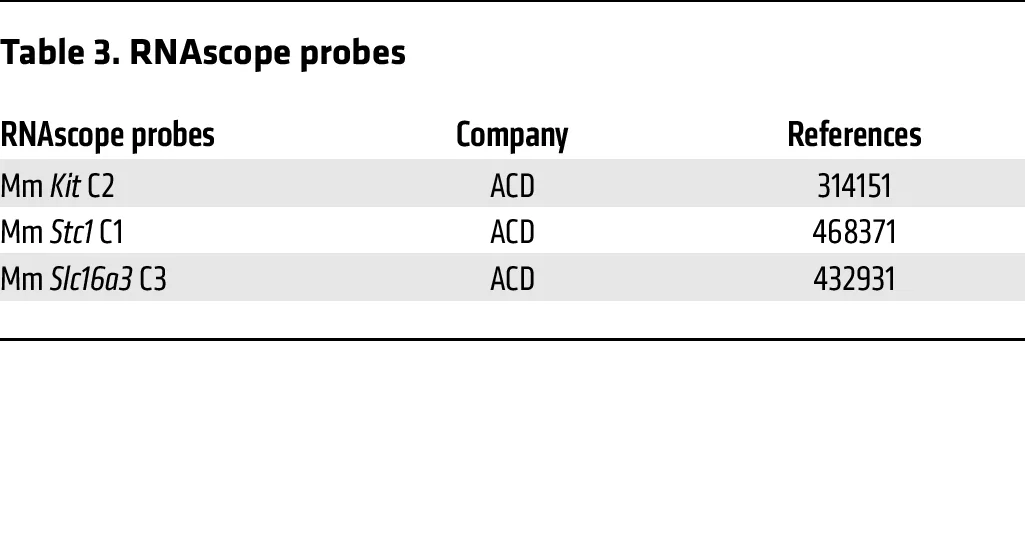
RNAscope probes

**Table 2:**
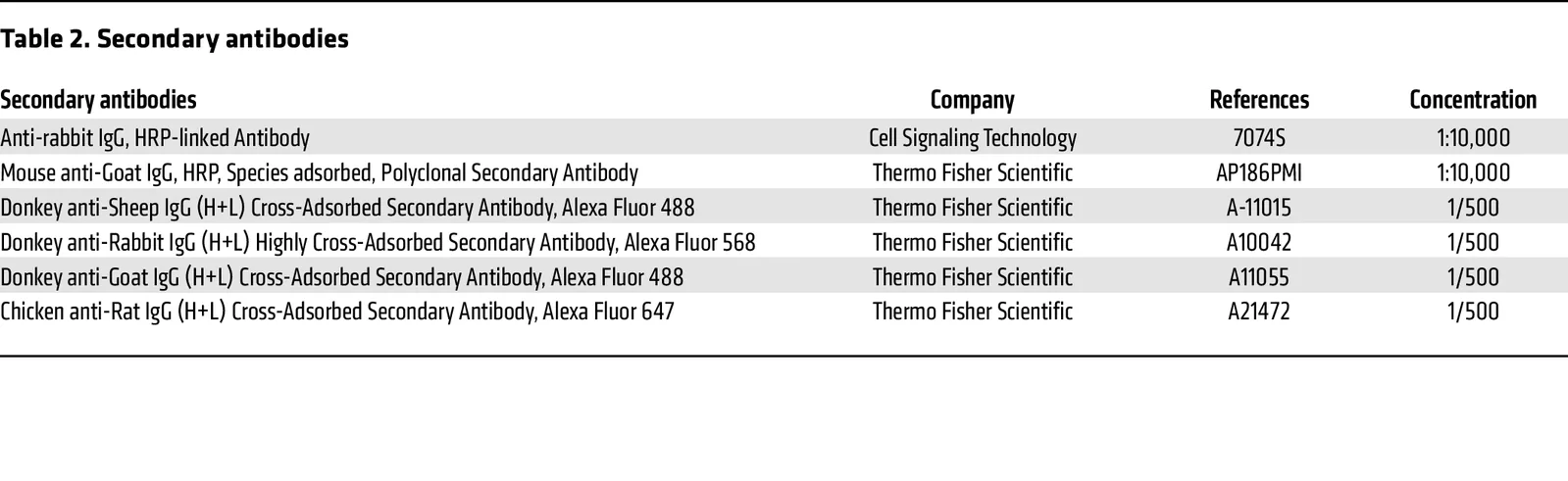
Secondary antibodies

**Table 1:**
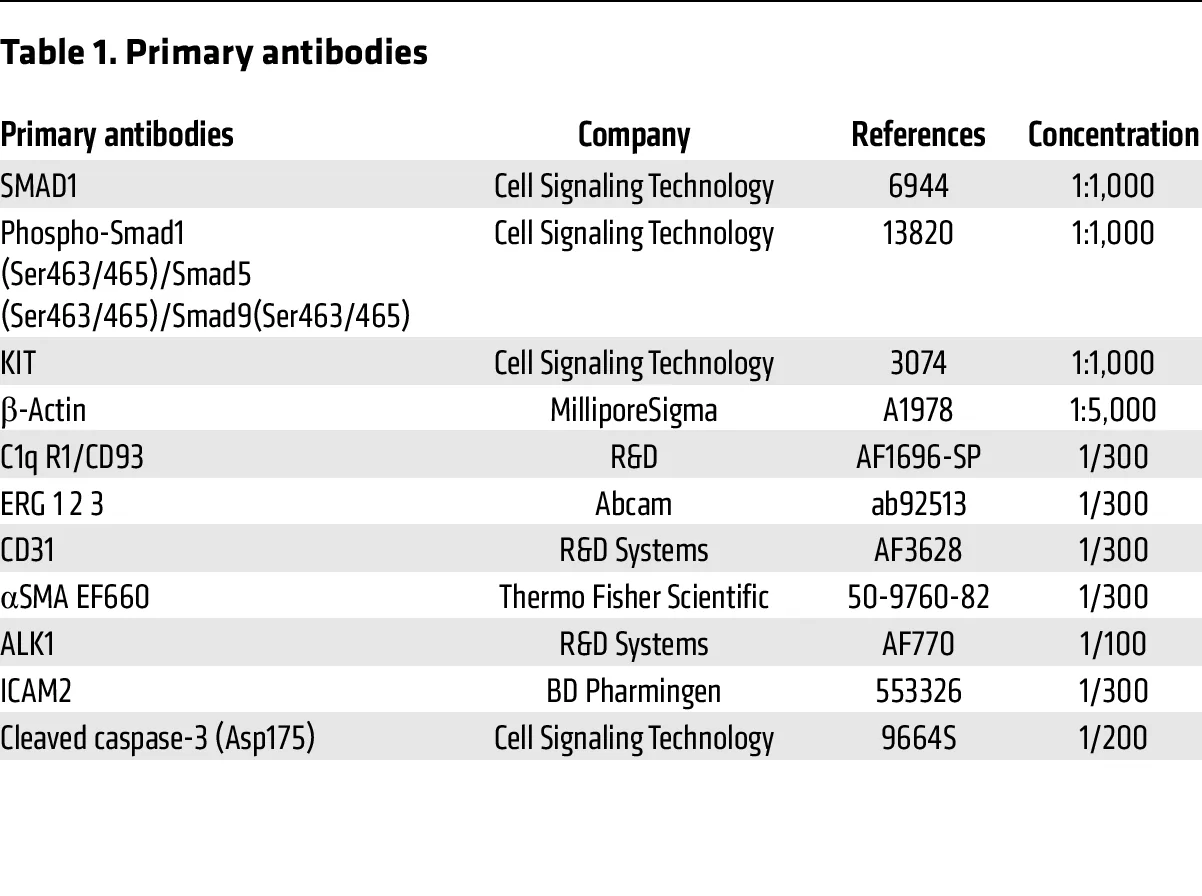
Primary antibodies

## Version 1 08/11/2026

In-Press Preview

## Version 2 10/01/2026

Electronic publication
