# Supplementary material for: Targeting KIT prevents brain arteriovenous malformations driven by ALK1-deficient angiogenic endothelial cells
Source: J Clin Invest. 2026 Aug 11;136(19):e197587. doi: 10.1172/JCI197587 (PMC13626853; doi:10.1172/JCI197587)

Full unedited blot/gel for Figure 5A

HUVEC Western Blot

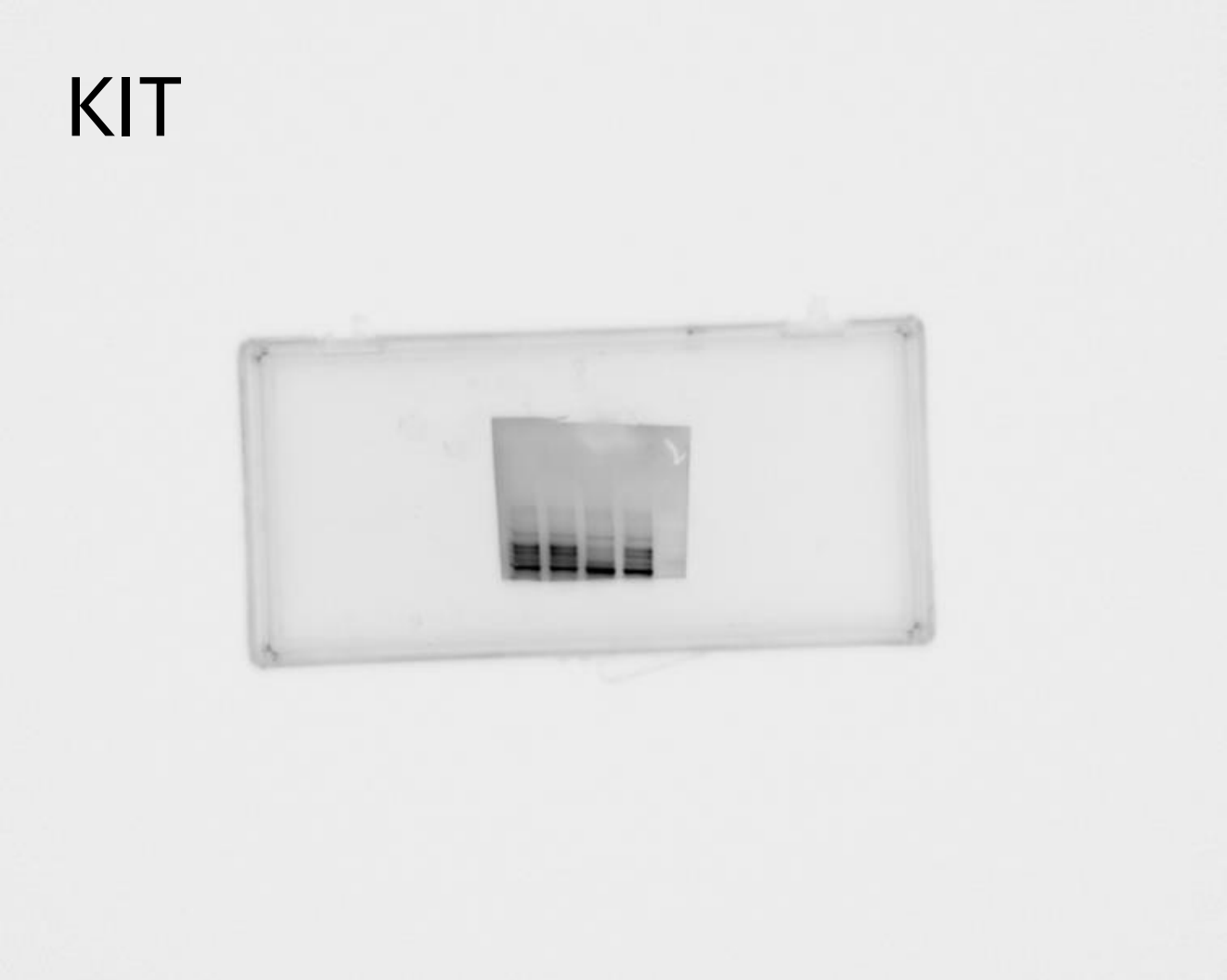

Colorimetrique

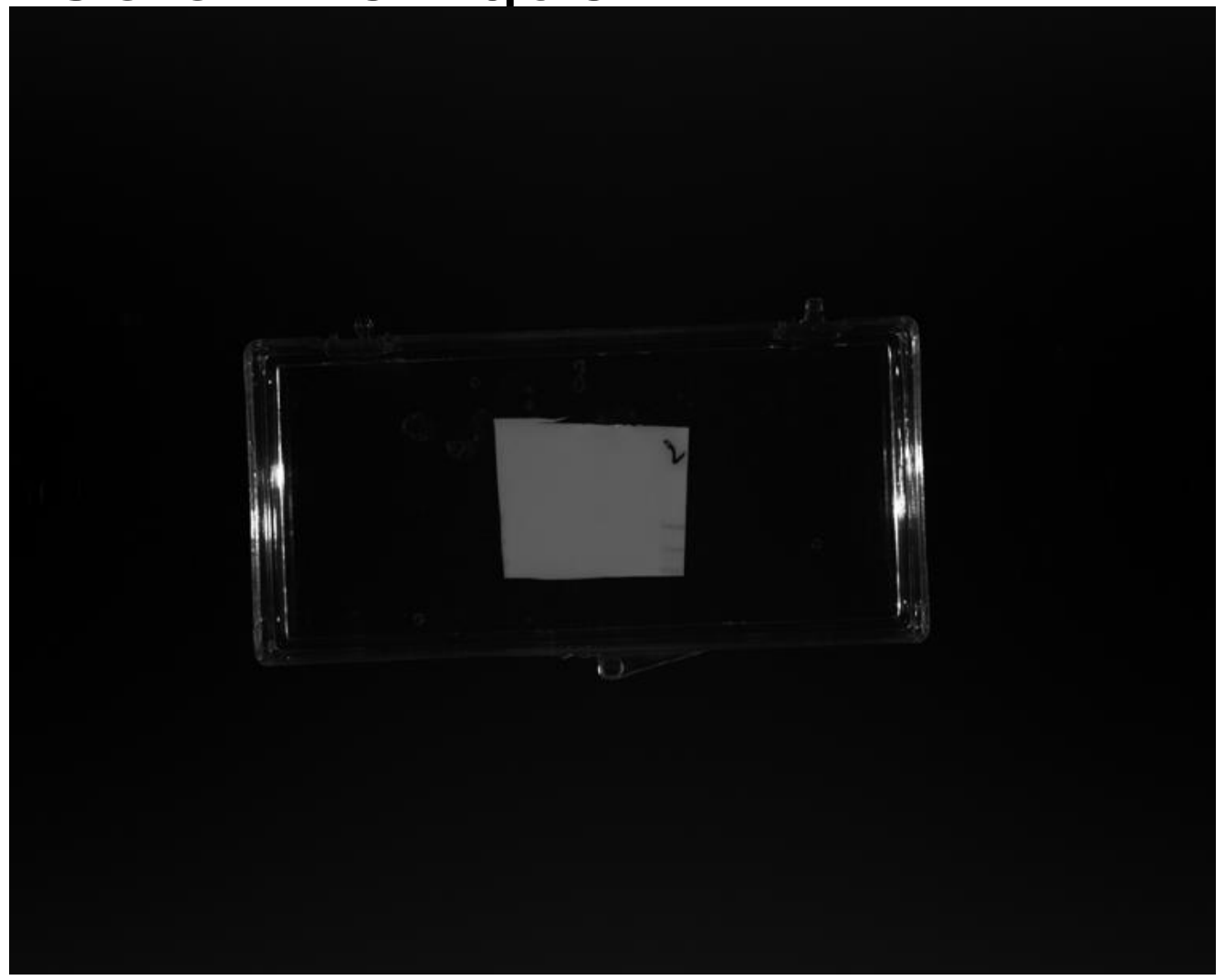

Merge

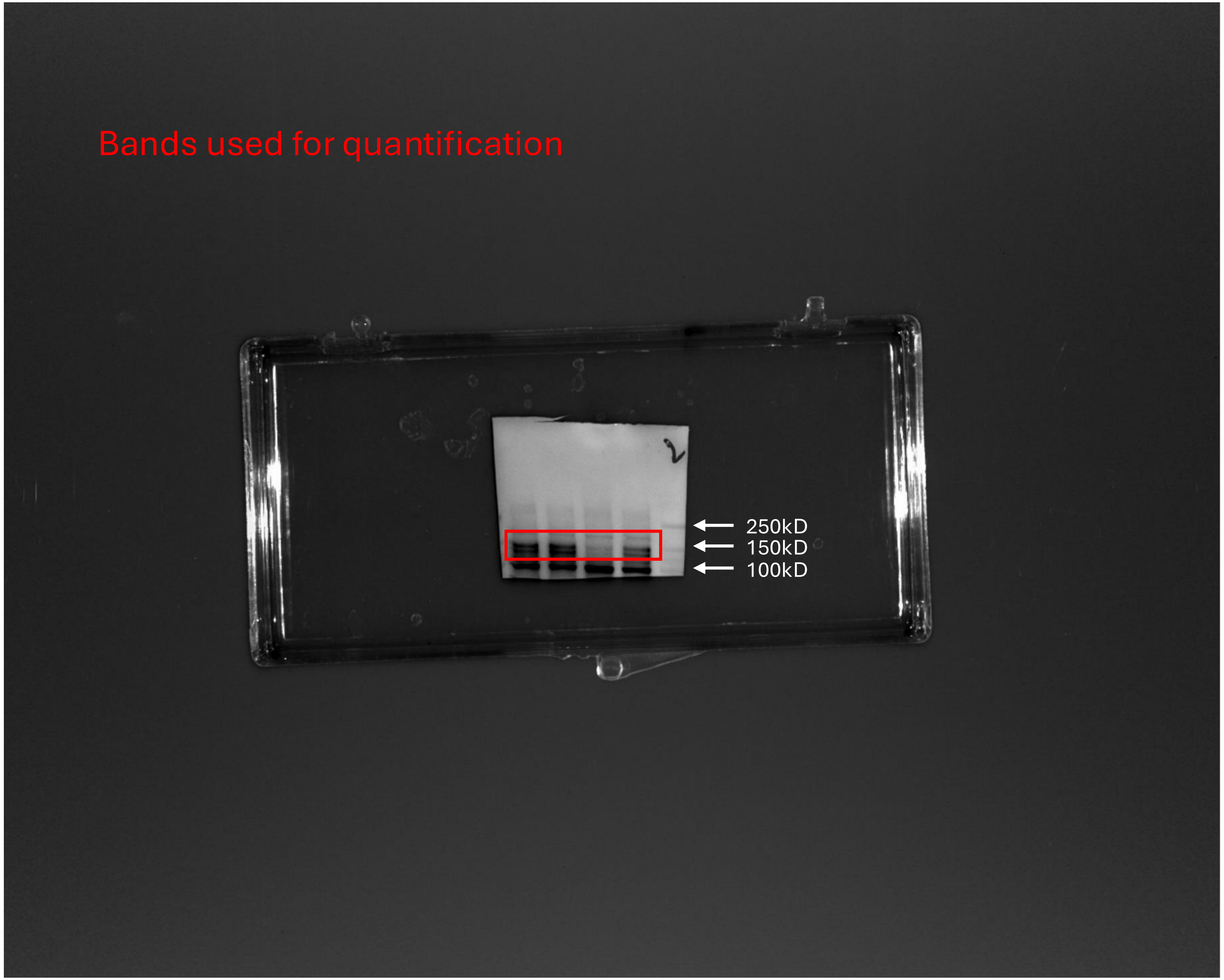

Full unedited blot/gel for Figure 5A

HUVEC Western Blot

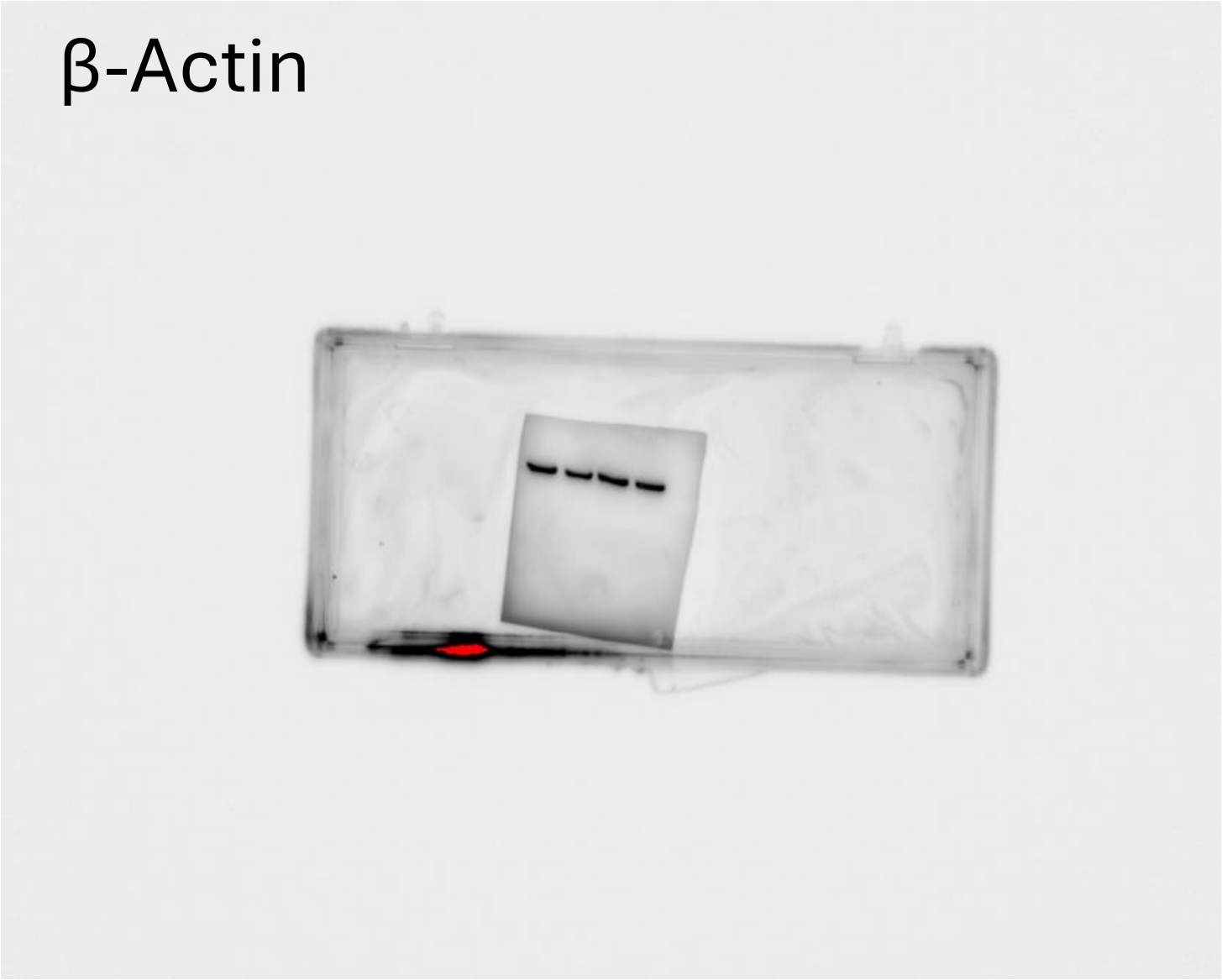

Colorimetrique

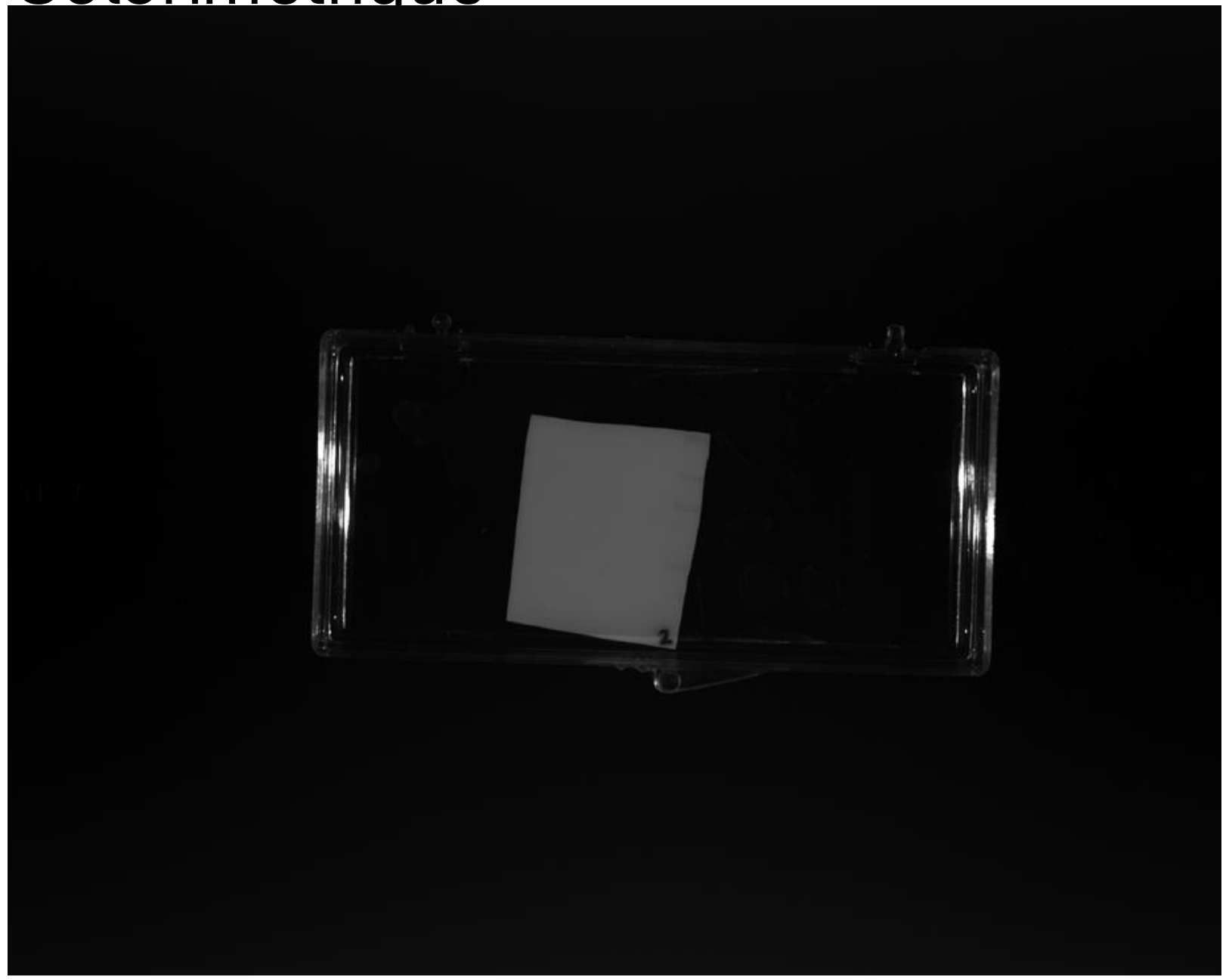

Merge

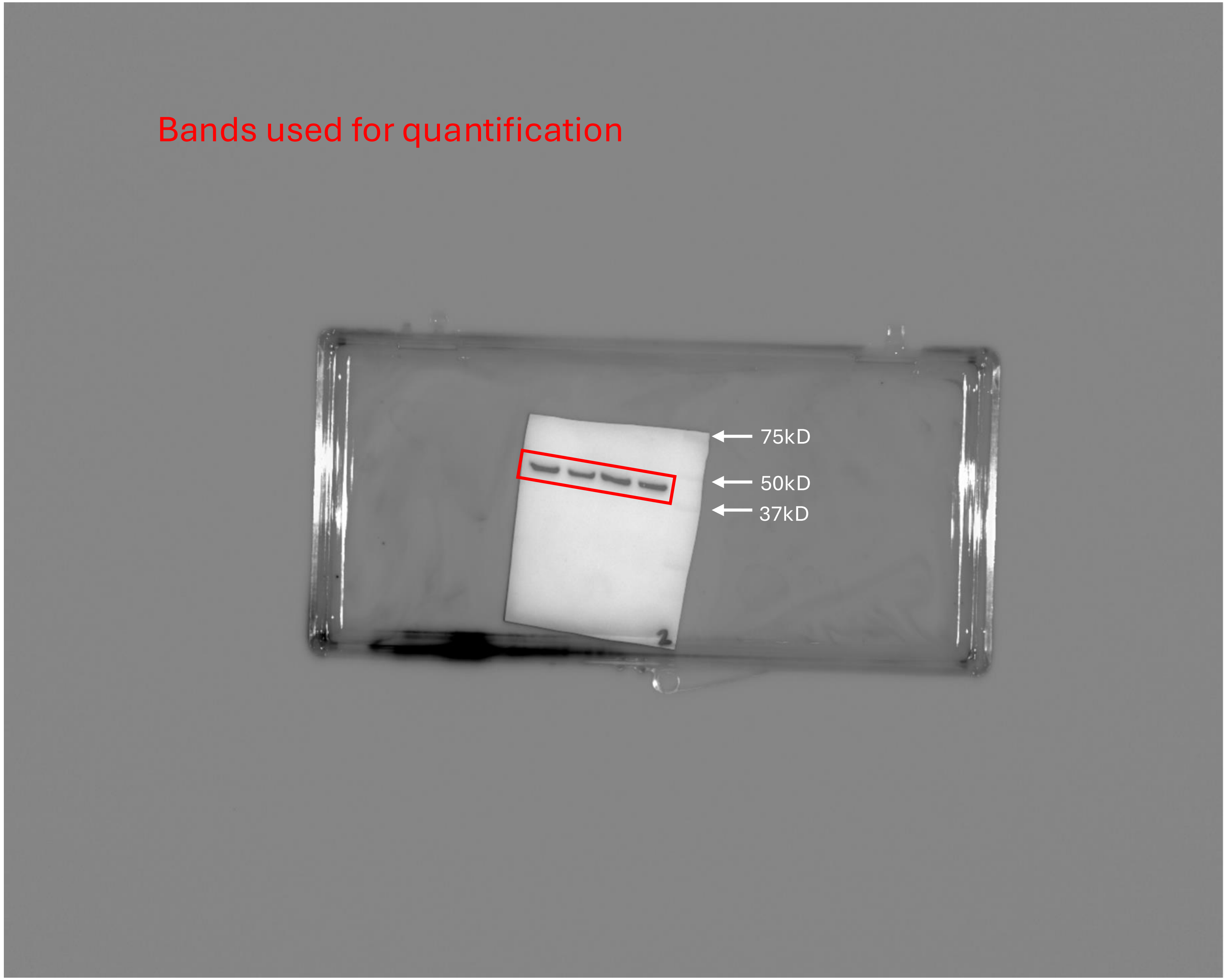

Full unedited blot/gel for Figure 5A

HUVEC Western Blot

pSMAD159

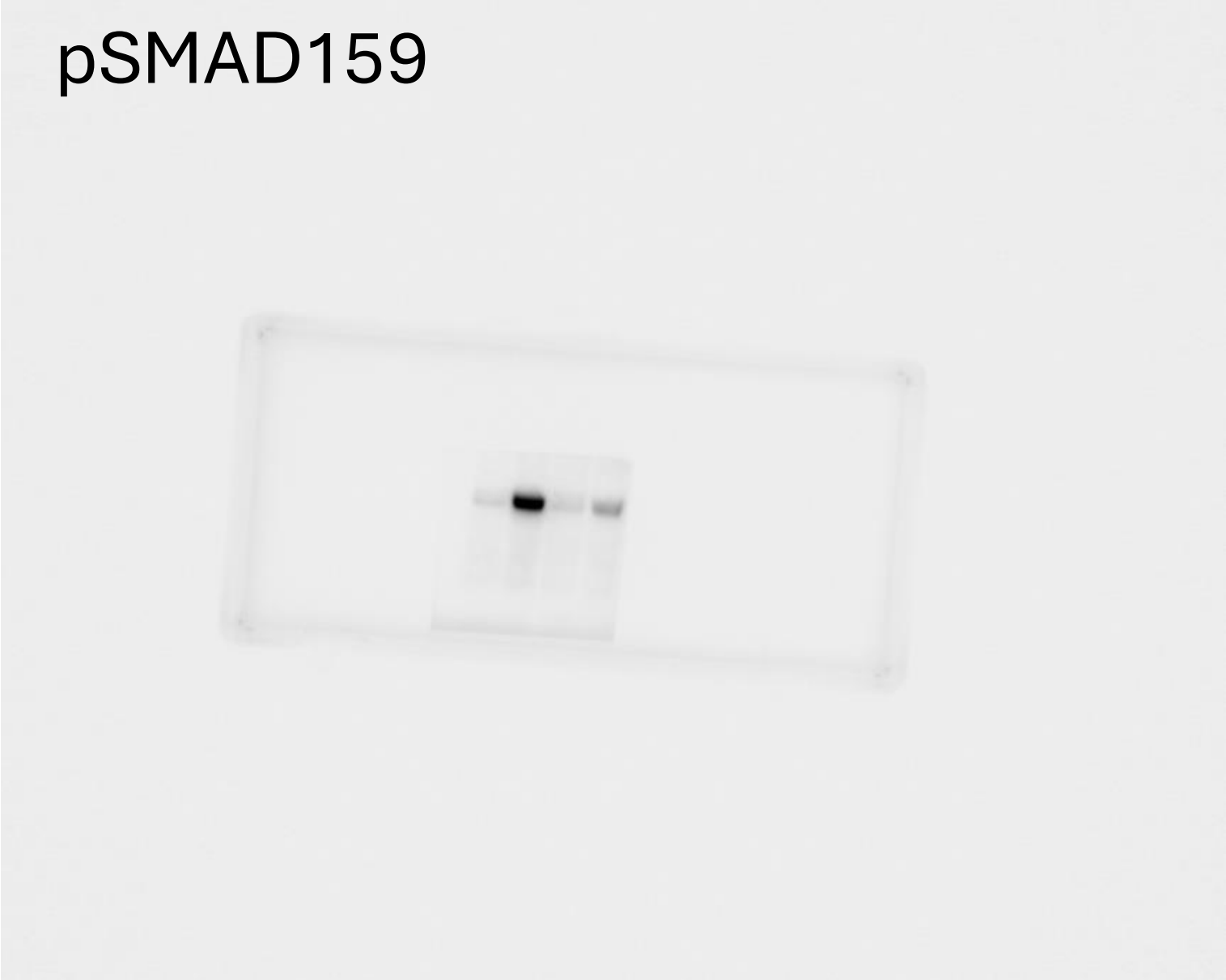

Colorimetrique

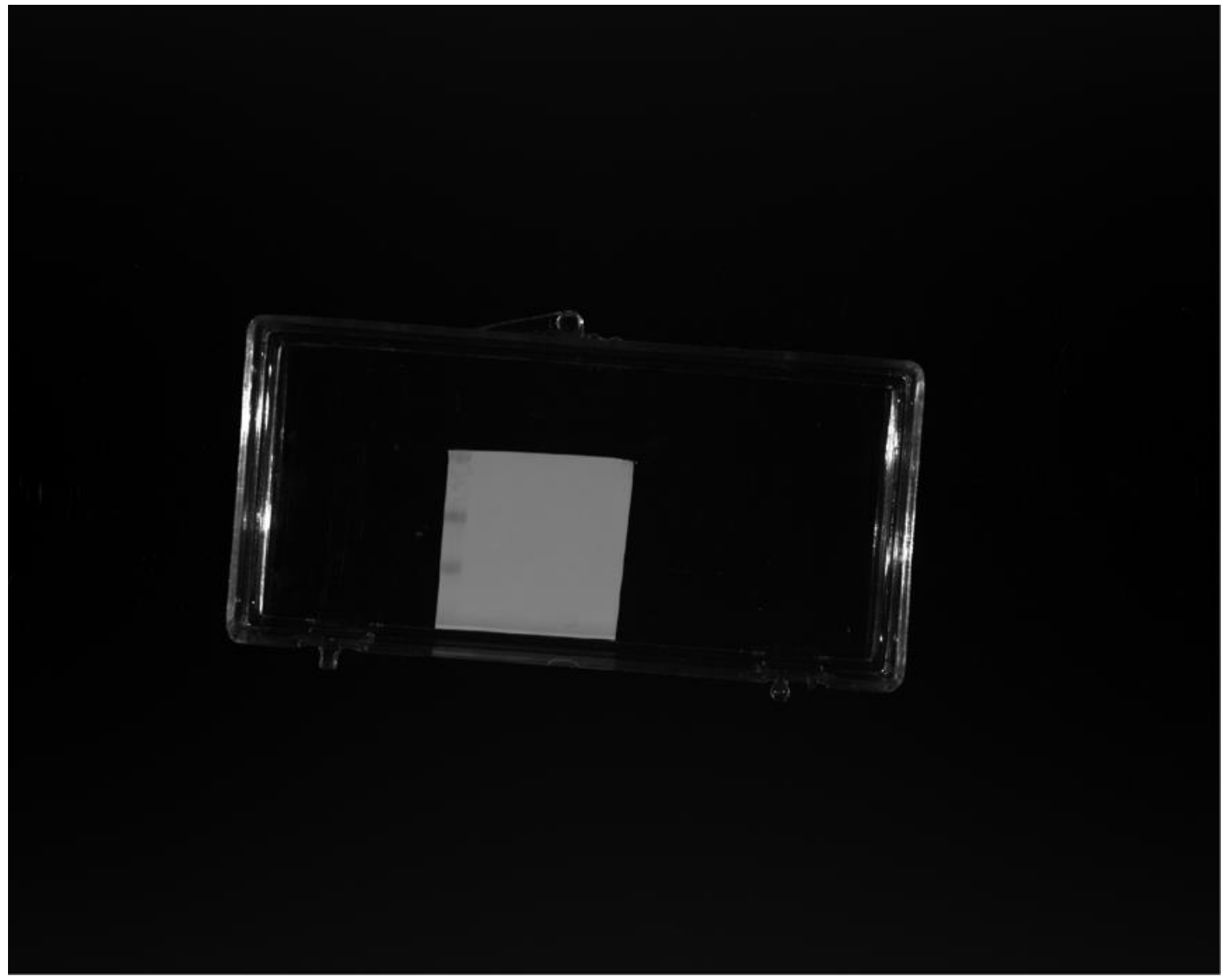

Merge

Bands used for quantification

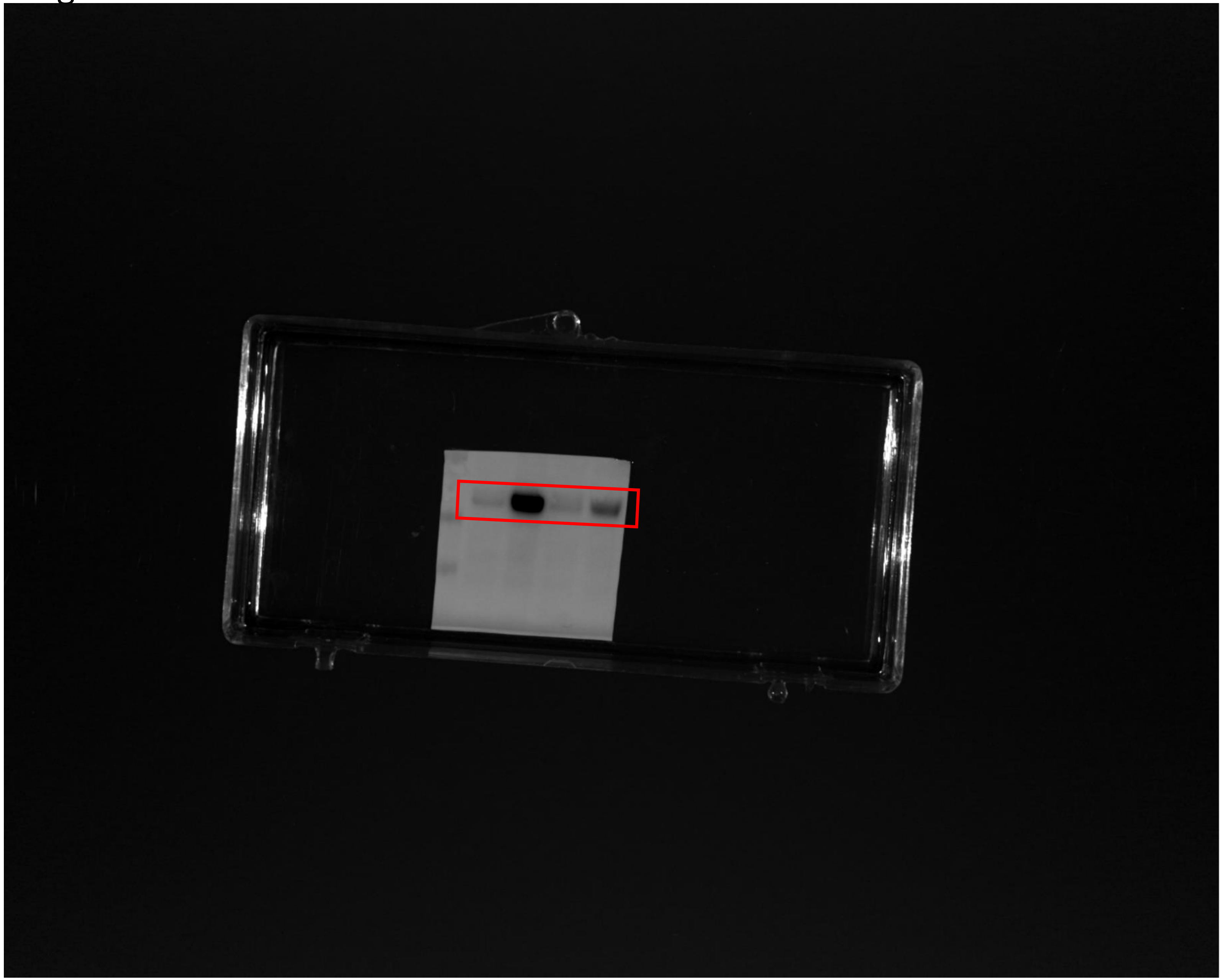

Full unedited blot/gel for Figure 5A

HUVEC Western Blot

Total smad1

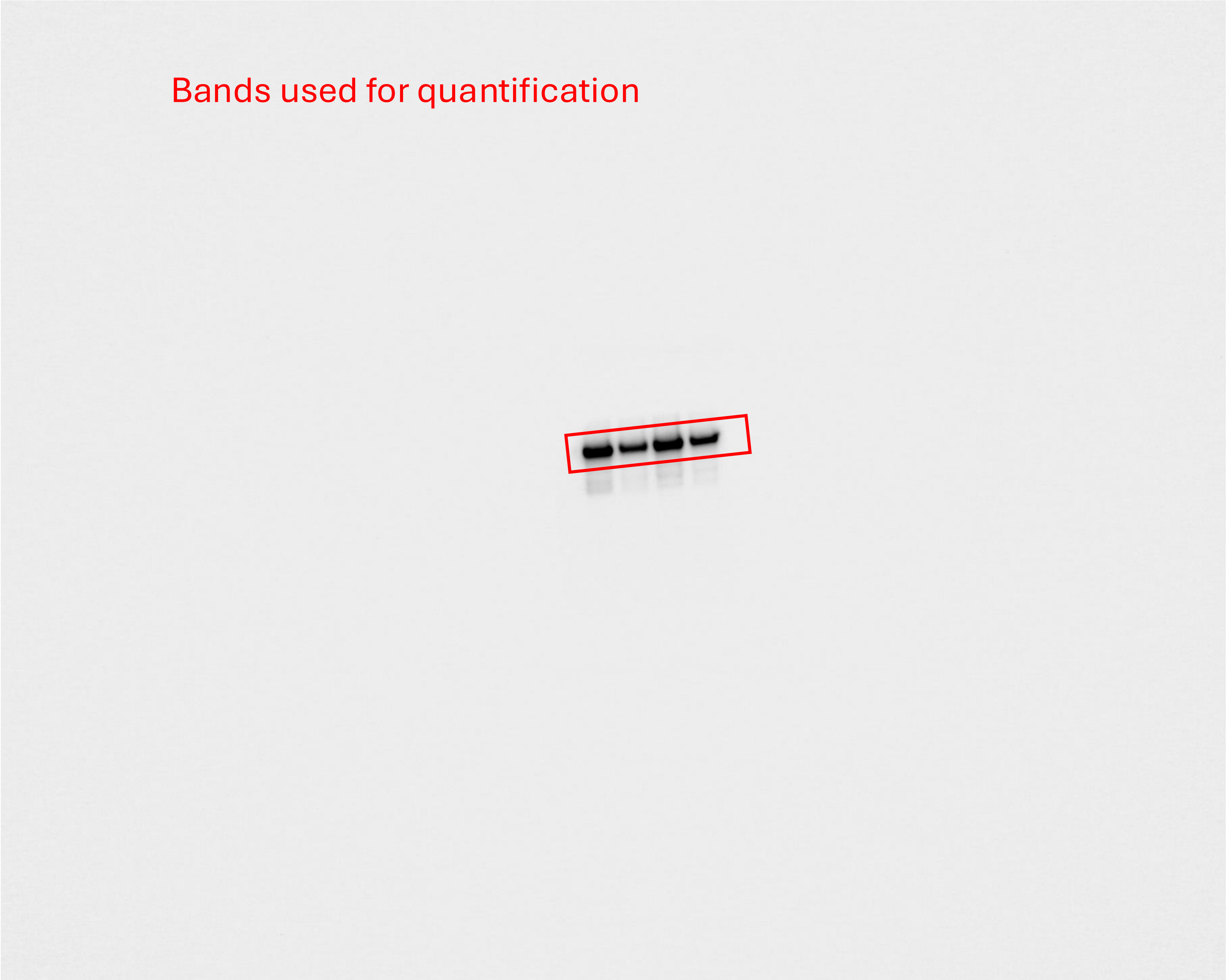

Full unedited blot/gel for Figure 5D

HBEC Western Blot

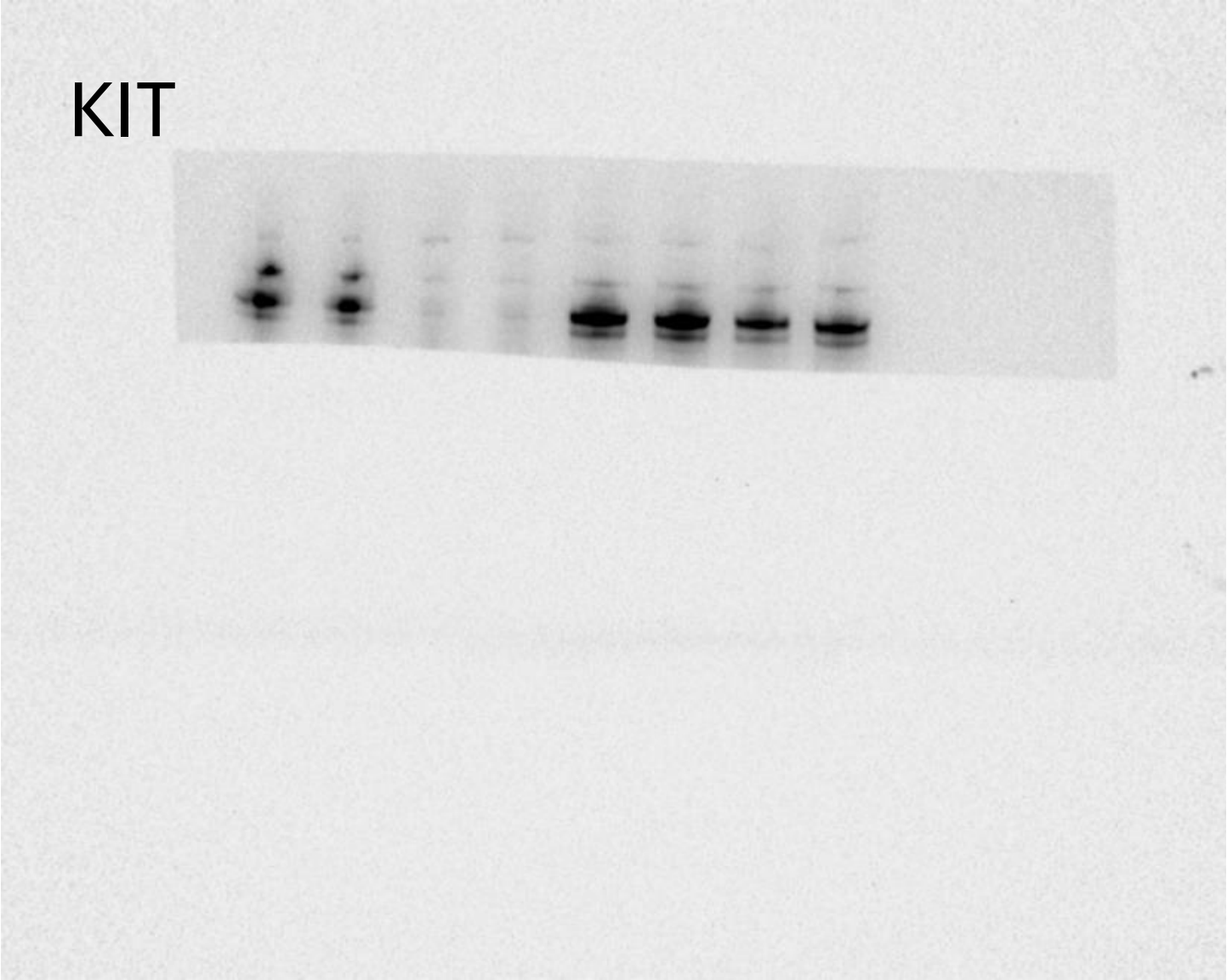

Colorimetrique

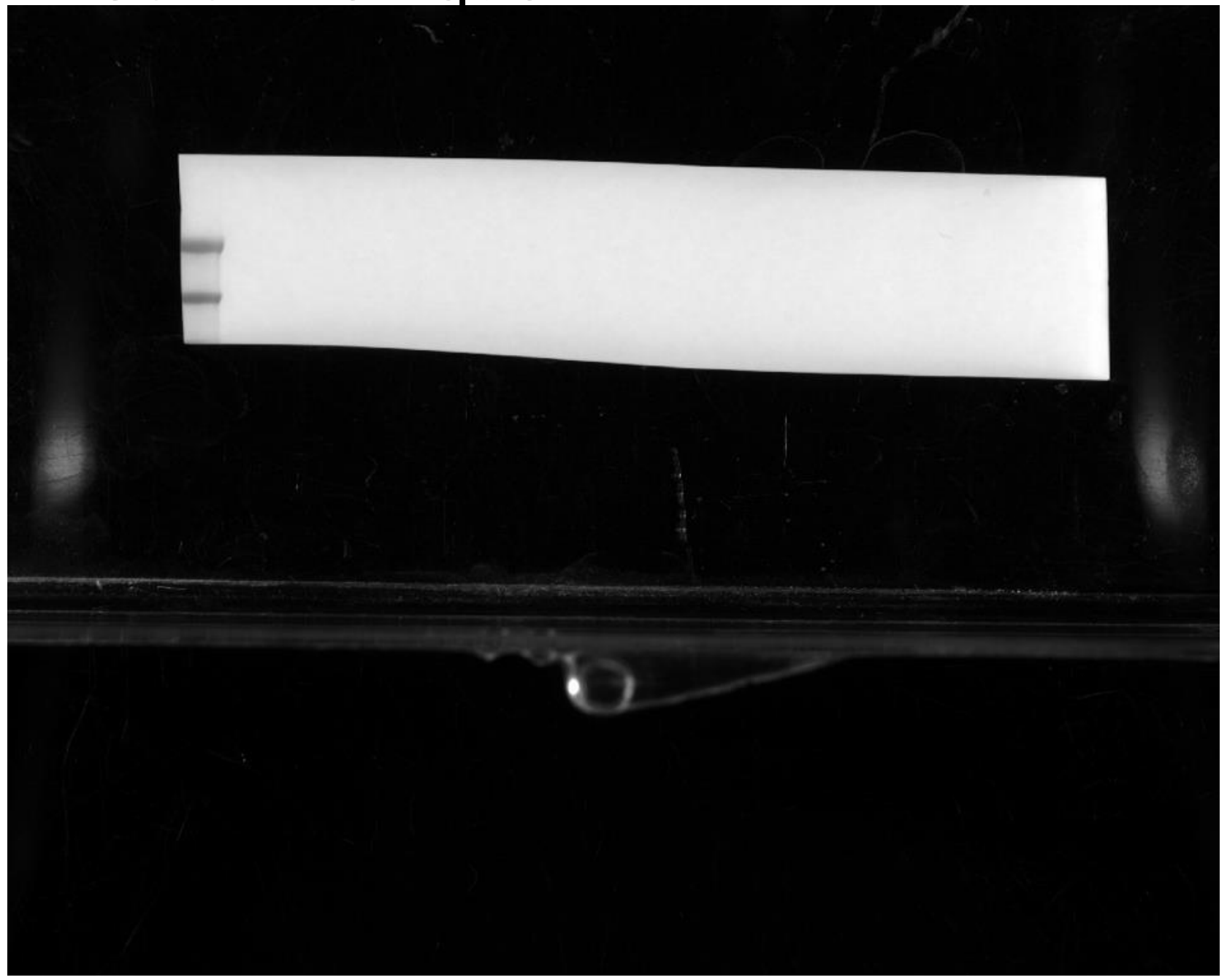

Merge

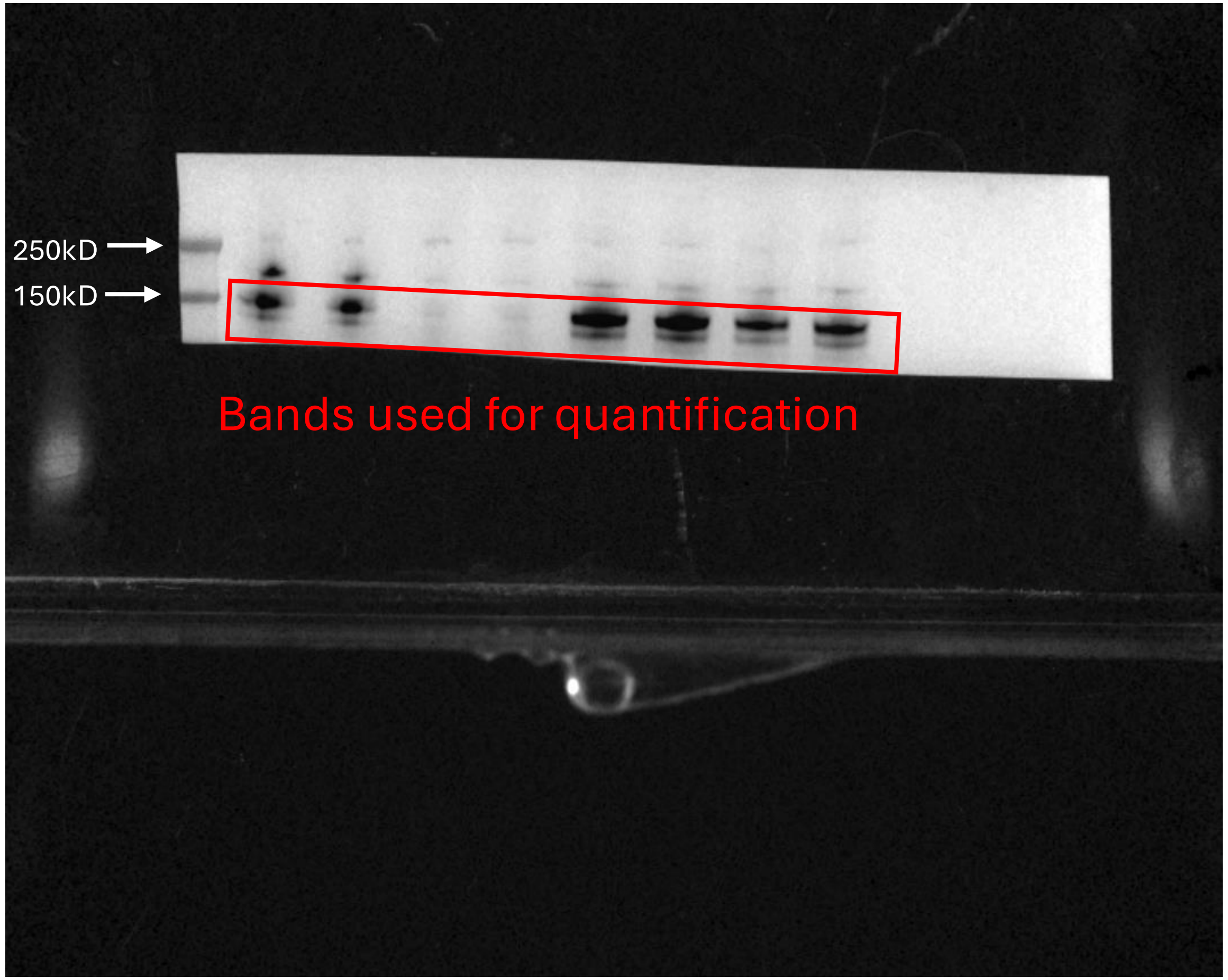

Full unedited blot/gel for Figure 5D

HBEC Western Blot

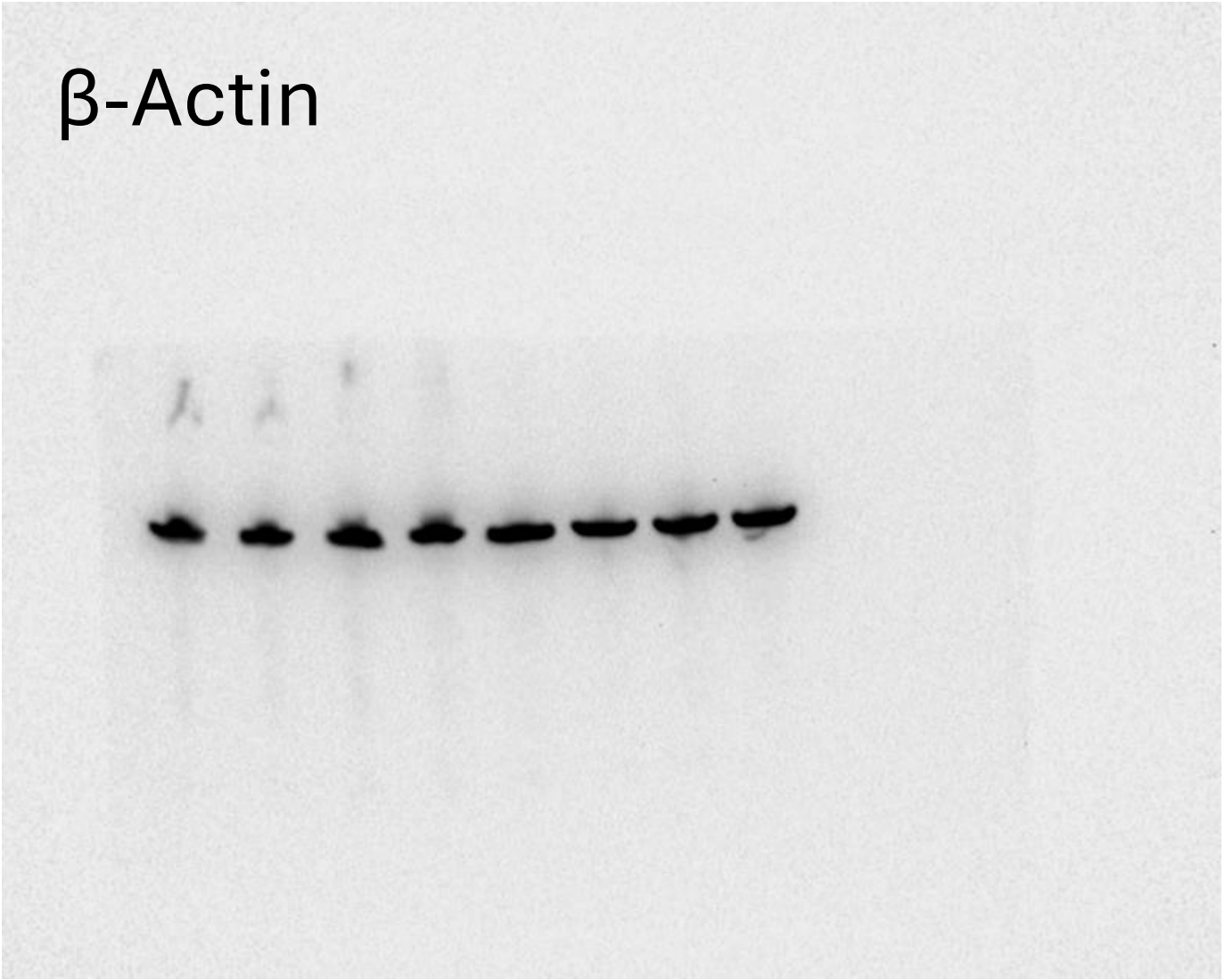

Colorimetrique

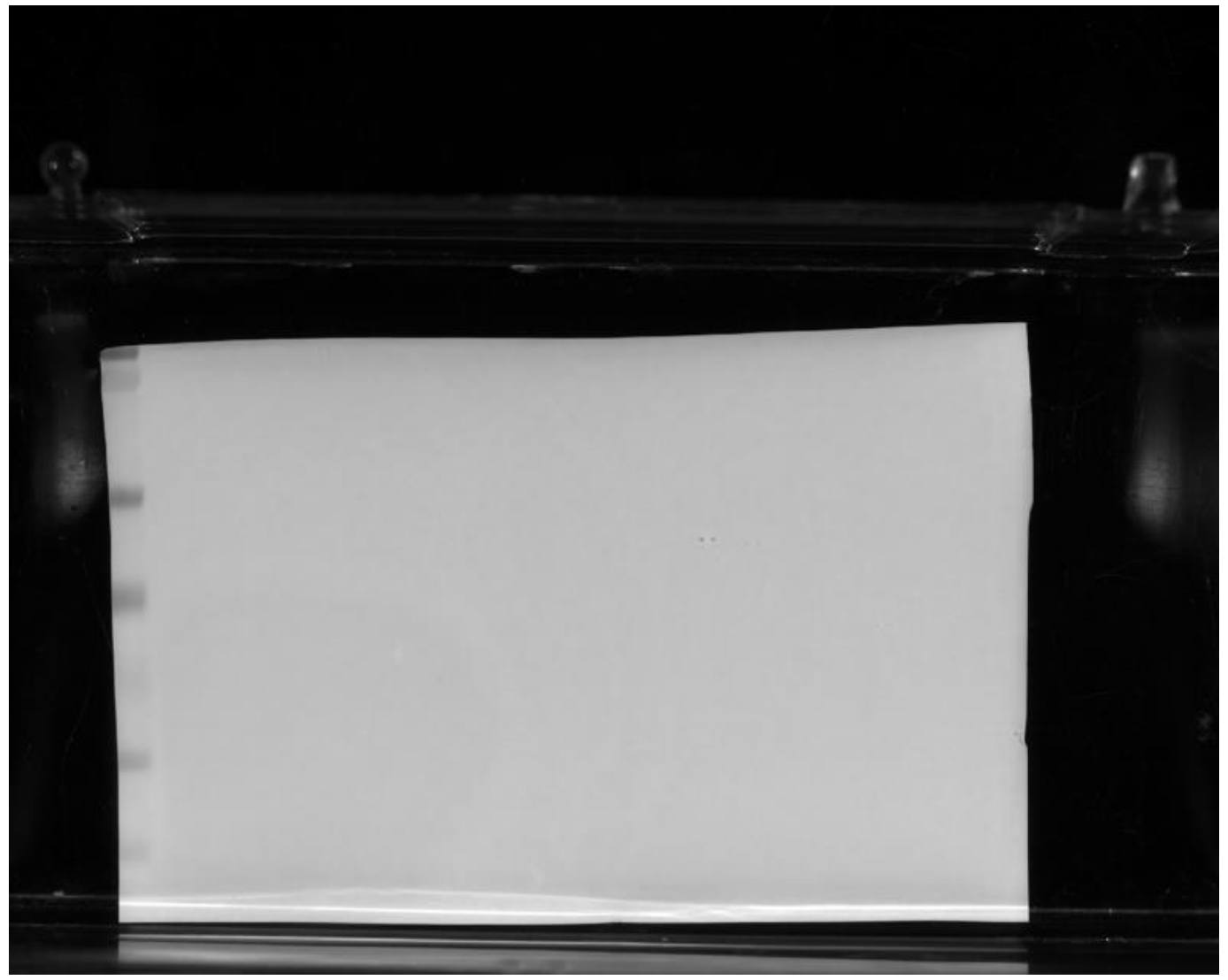

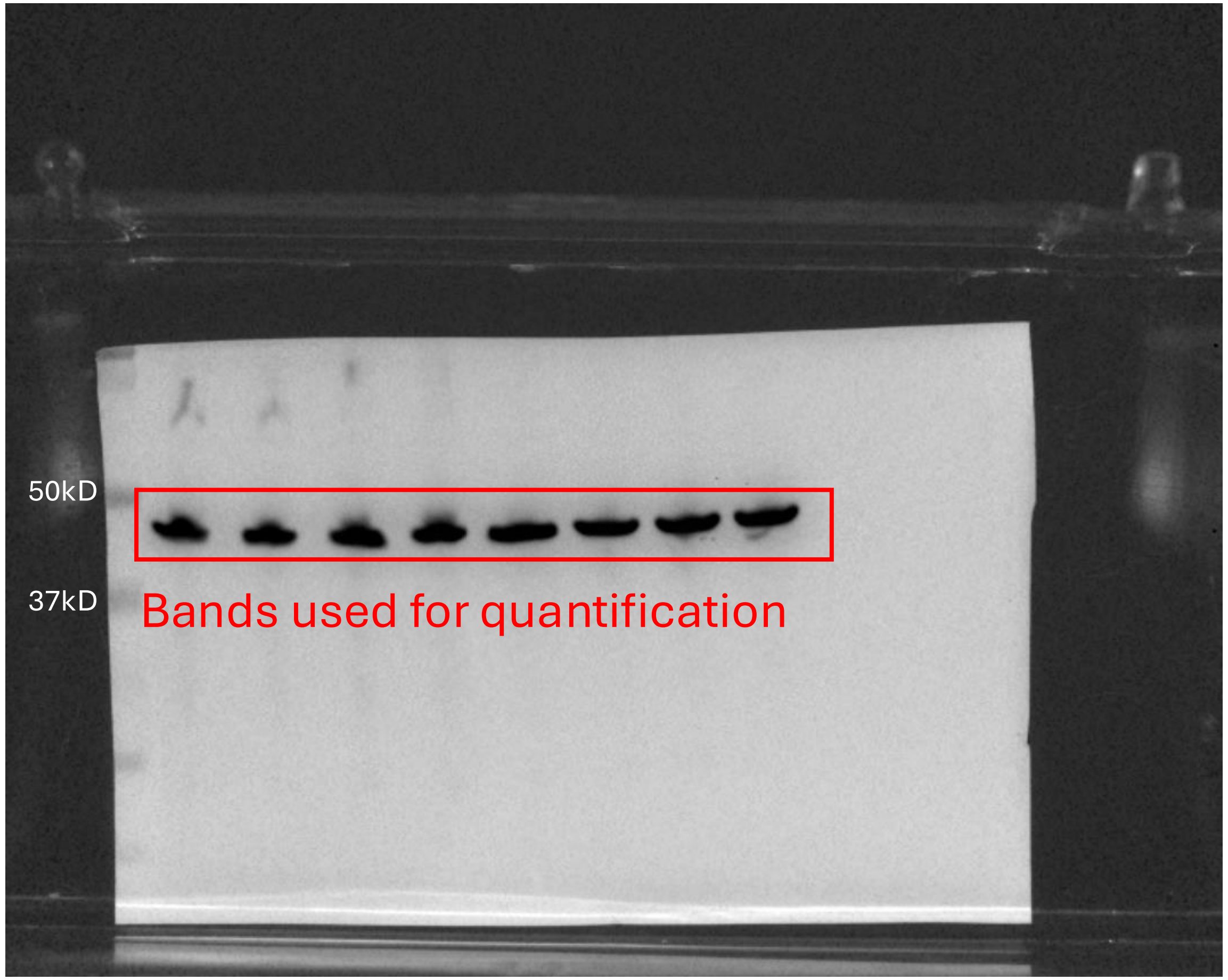

Full unedited blot/gel for Figure 5D

HBEC Western Blot

pSMAD159

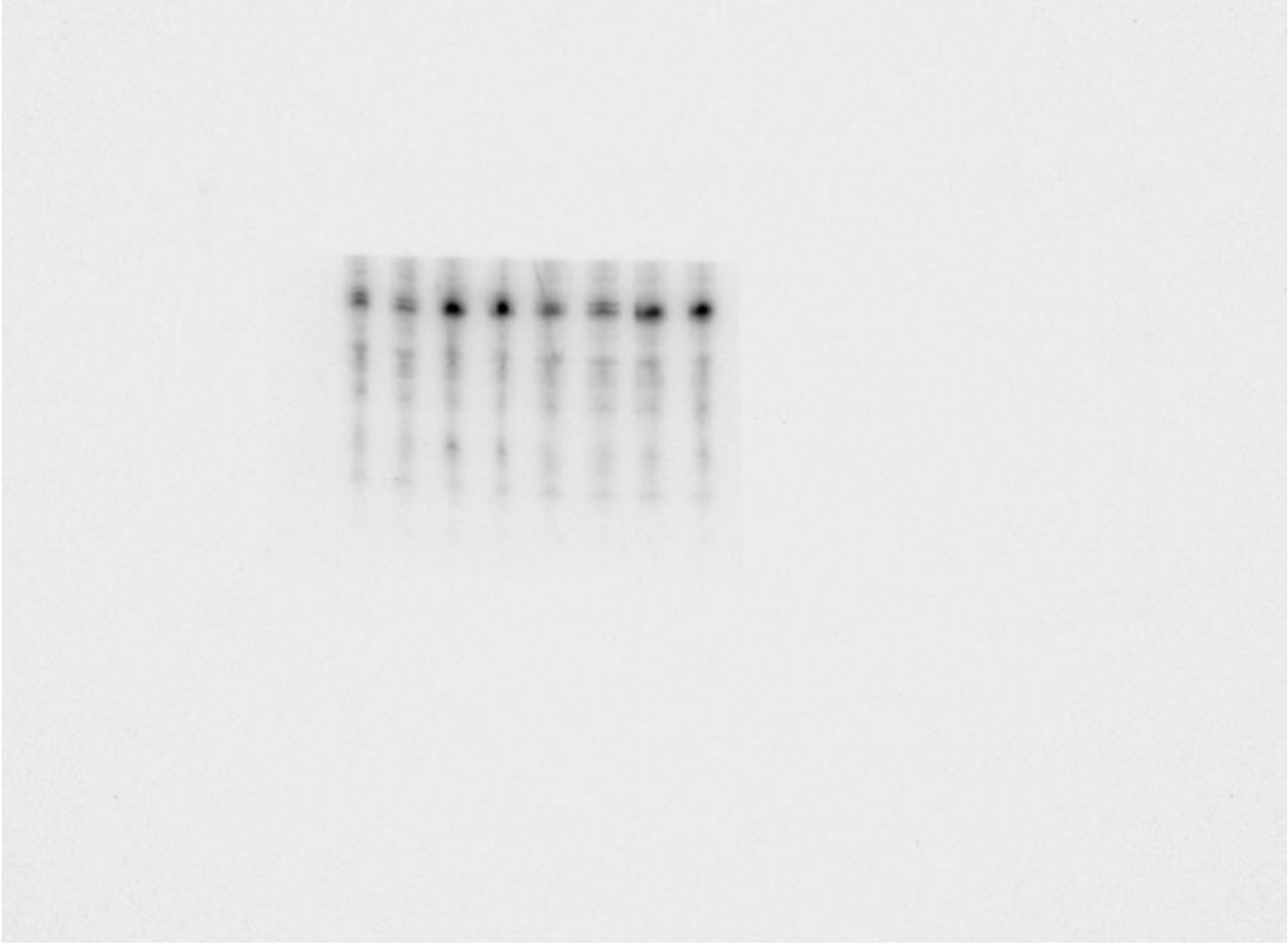

Colorimetrique

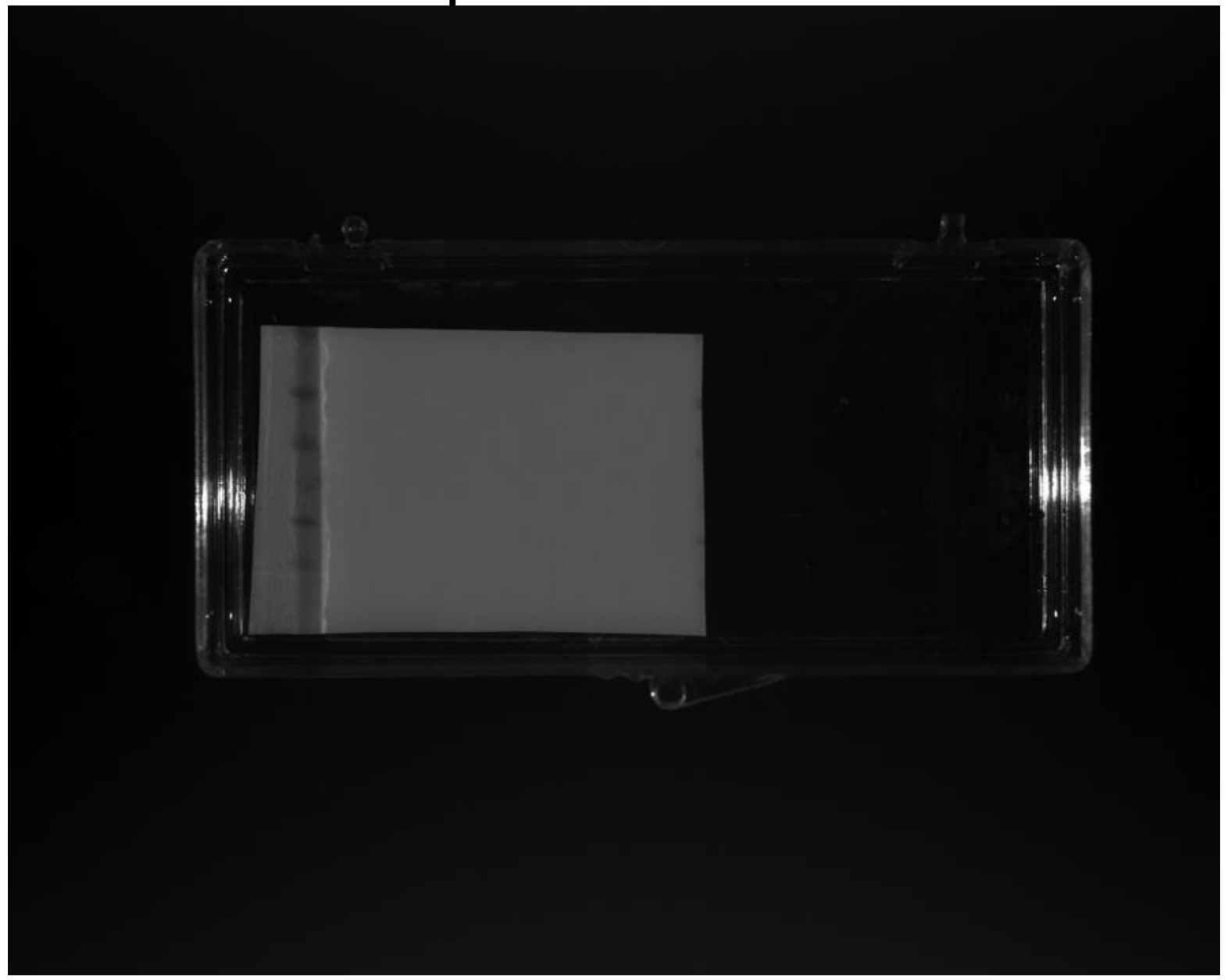

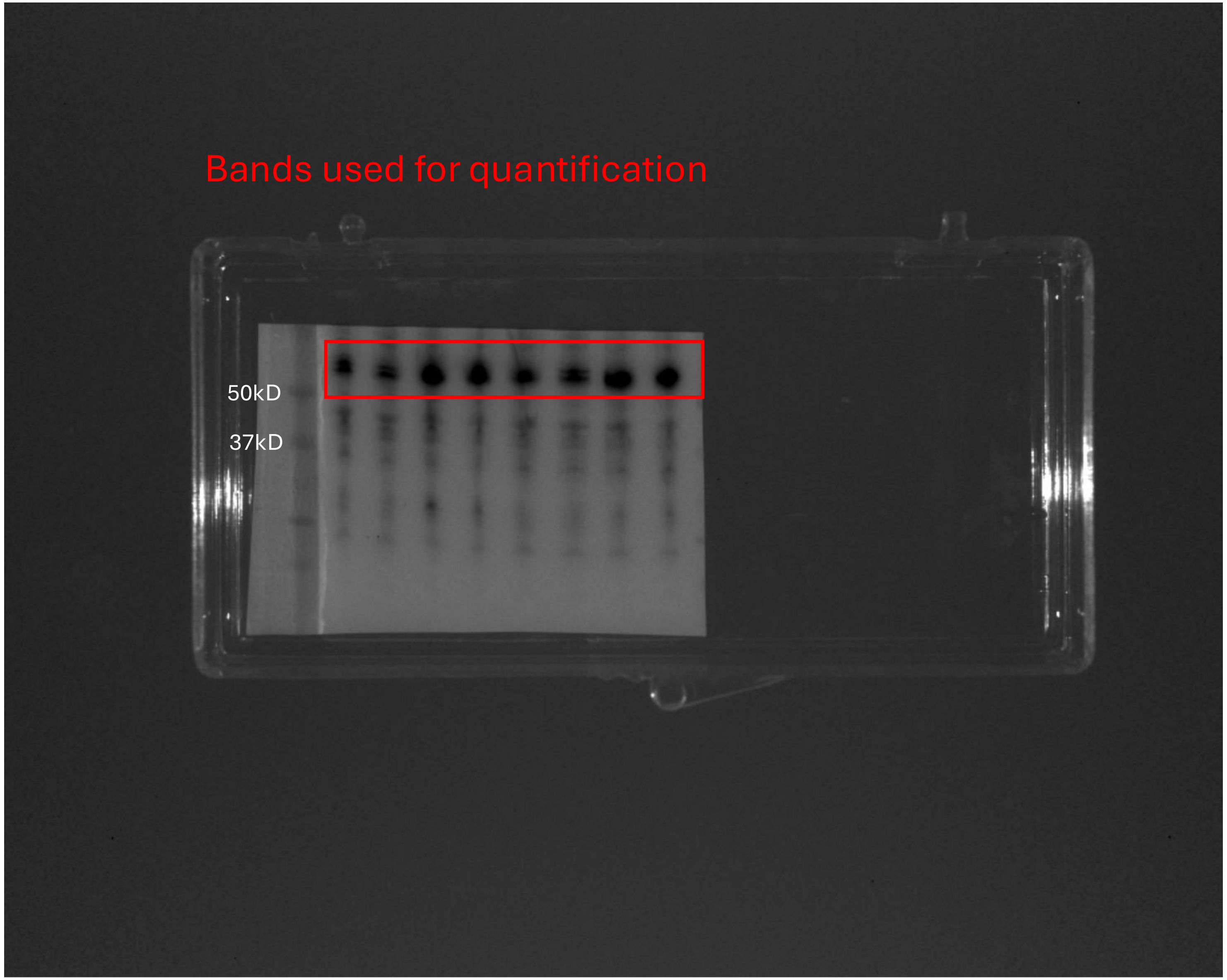

Full unedited blot/gel for Figure 5D

HBEC Western Blot

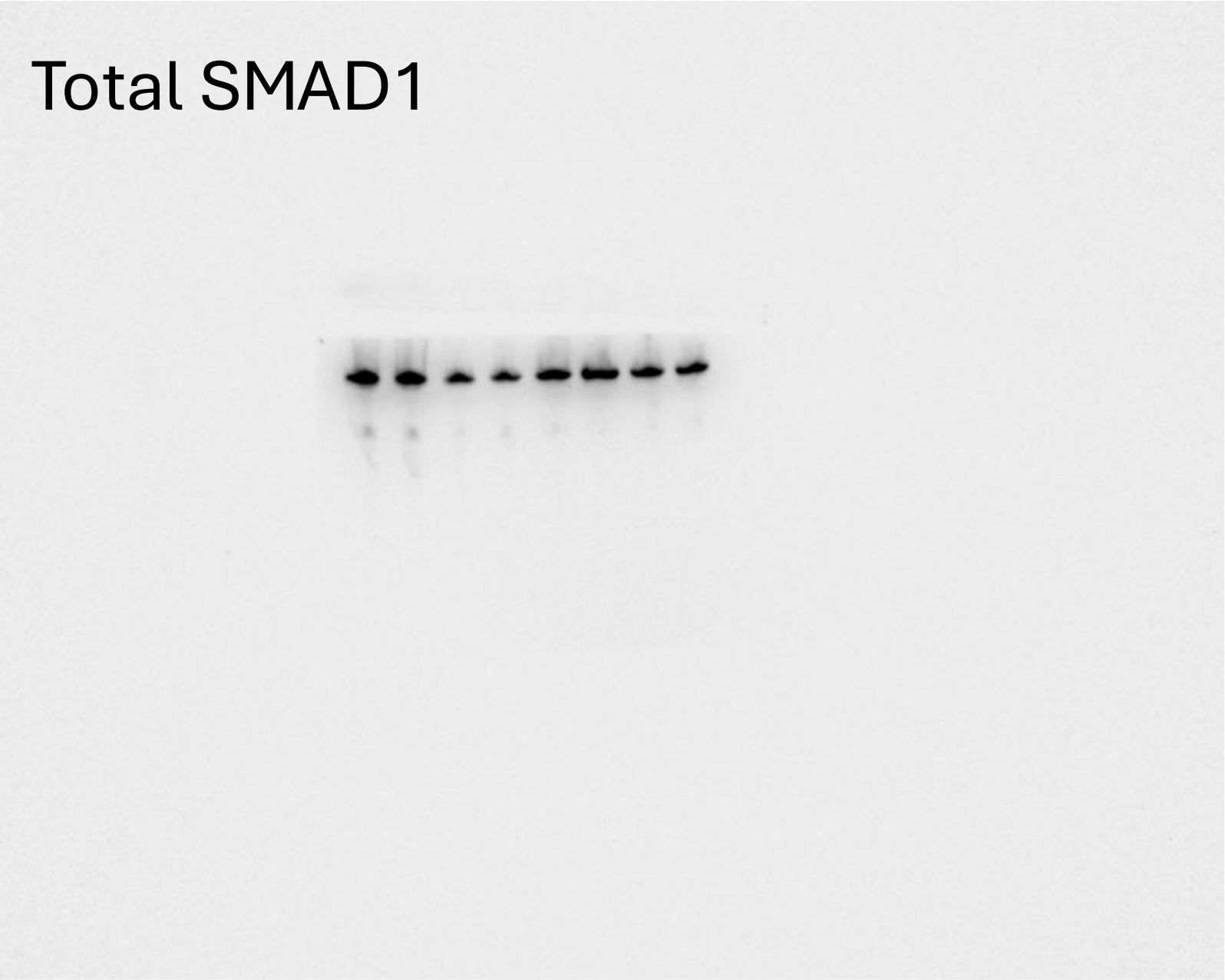

Colorimetrique

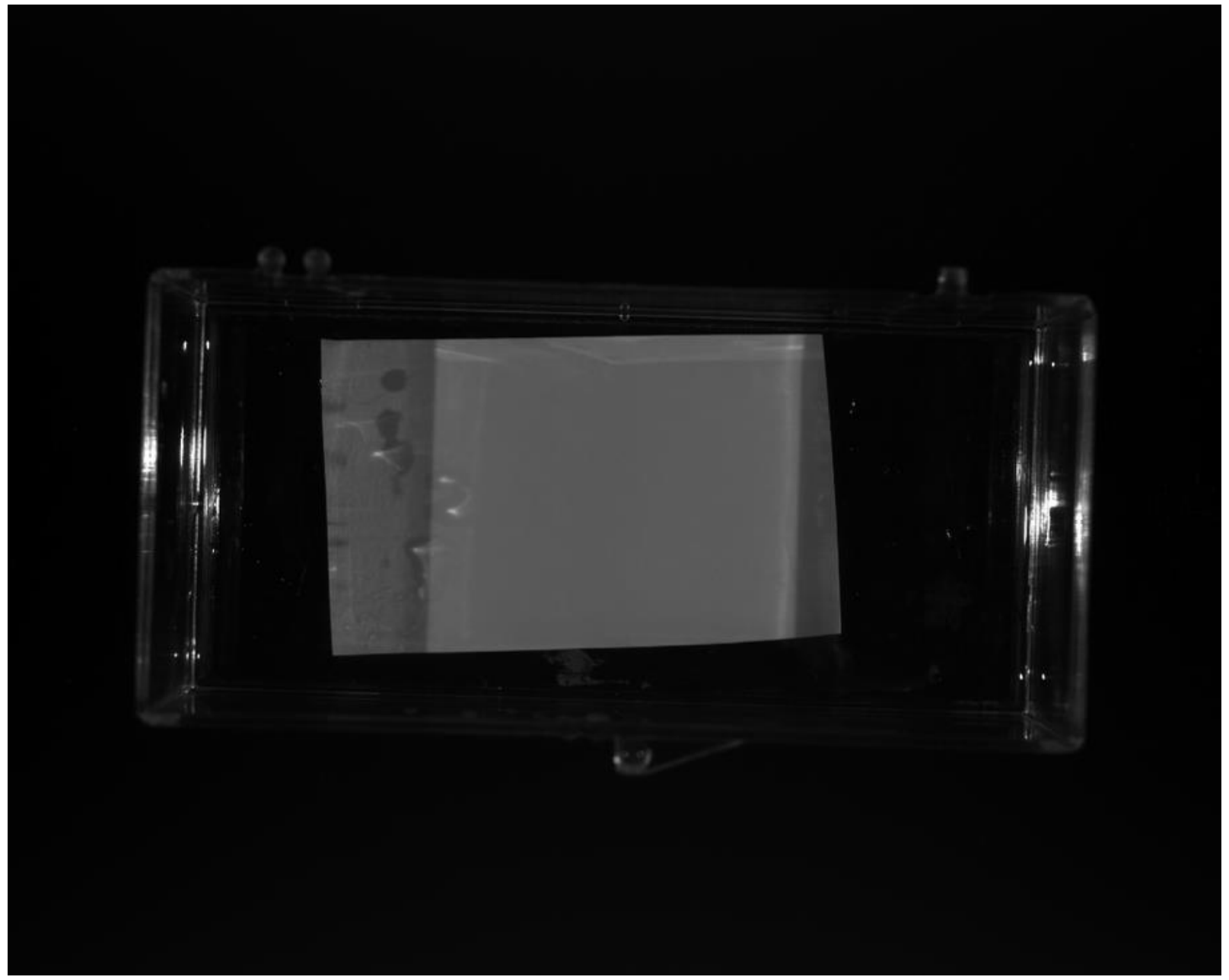

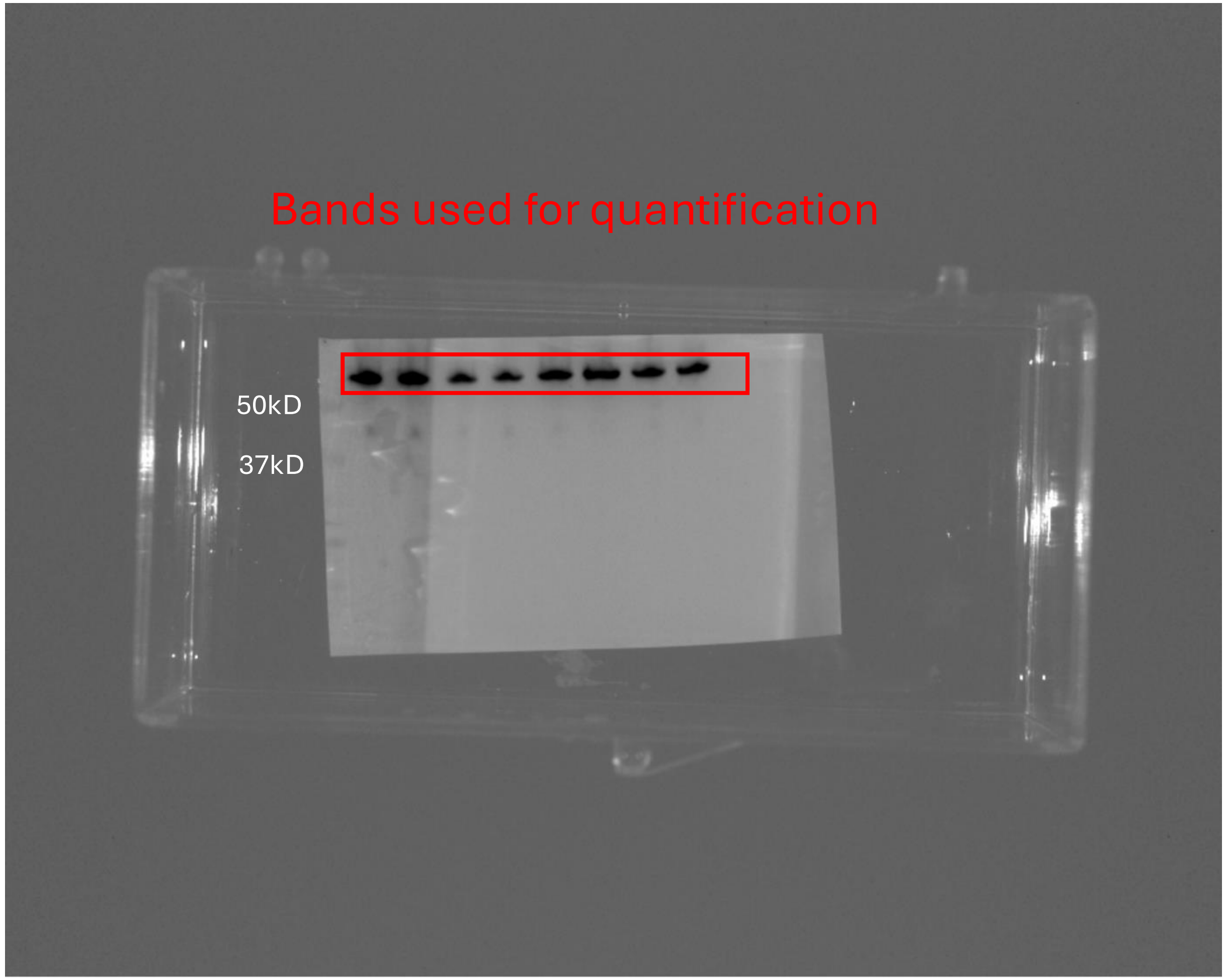

Supplement: Supplementary file 1 [file jci-136-197587-s310.pdf]
